# Parameter-Independent Feature Ranking with Volume-Integrated Sharma–Mittal Entropy: Kernel-Based Estimation, Theoretical Properties and Empirical Validation

**DOI:** 10.3390/e28080933

**Published:** 2026-08-20

**Authors:** Nida Oruç Ünal, Muzaffer Göztaş, Doğan Yıldız

**Affiliations:** Department of Statistics, Faculty of Arts and Sciences, Yıldız Technical University, 34000 Istanbul, Türkiye; muzaffer.goztas@yildiz.edu.tr (M.G.); dyildiz@yildiz.edu.tr (D.Y.)

**Keywords:** feature selection, Sharma–Mittal entropy, kernel density estimation, information gain, rank correlation, continuous variables

## Abstract

Feature selection is a critical step in regression problems where a large number of continuous explanatory variables explain the same target through different dependency structures. Classical filters may remain sensitive to a single form of dependence, a single scale, or a specific discretization scheme; generalized entropy measures, on the other hand, typically require the parameters to be fixed at a single point. This study proposes a framework that evaluates the Sharma–Mittal entropy volumetrically across a two-dimensional parameter region rather than for a single parameter pair. For the continuous target and explanatory variables, the marginal, joint, and conditional densities are obtained using a Gaussian kernel density estimation; the conditional entropy and information gain surfaces are integrated across the region Ω = [0.05, 0.95]^2^ in the *α*-*β* plane to define three indices: PICSME, which measures the conditional uncertainty volume; PIGSME, which measures the gain volume; and NIGSME, which is the ratio of this gain to the total entropy volume of the target. The method is supported by bandwidth consistency and the renormalization of conditional densities; thus, the issue of negative gain that can occur in the continuous variables is resolved, yielding positive and interpretable scores across all six datasets. It is formally demonstrated that the fact that the three indices produce the same ranking is not an empirical observation but rather the result of a monotonicity relationship valid under a fixed target entropy volume. The method is compared with Pearson and Spearman correlations, the Shannon information gain, mutual information, and random forest variable importance across six regression datasets (Airfoil Self-Noise, AirQualityUCI, BodyFat, Meteorology, Concrete, and WineQualityWhite) that differ in their sample size, dimensions, and application domain. The evaluation is not limited to ranking consistency; the out-of-sample prediction performance is measured using least-squares models on the top-k subsets, with rankings calculated from the training partition. The findings show that NIGSME exhibits a performance comparable to that of built-in filters, outperforms them on the Concrete and Meteorology datasets, and never ranks as the weakest method on any dataset. The results demonstrate that volumetric entropy metrics defined across the entire parameter space provide a feature-ranking tool that is independent of parameter selection for continuous variables.

## 1. Introduction

Feature selection is one of the strategic components of the modeling process, particularly in regression problems where a large number of predictors explain the same target variable with varying strengths and different dependency structures. An effective filtering mechanism not only eliminates unnecessary variables but also reduces the computational cost of a model, minimizes the risk of overfitting, and enhances interpretability. Therefore, feature selection serves the combined objectives of improving predictive performance and computational efficiency, and providing a better understanding of the data-generation mechanism [1,2,3,4].

Classical filtering approaches often rely on a Pearson correlation, which measures linear relationships [5]; Spearman’s rank correlation, which summarizes monotonic dependence [6]; information-theoretic criteria based on the reduction in uncertainty [7,8]; mutual information-based selection criteria [9,10,11,12,13]; or model-based importance measures, such as random forest variable importance [14,15]. While these methods are widely accepted, they may be sensitive to a single form of dependence, a single scale, or a specific discretization design. In particular, the fact that entropy and mutual information calculations for continuous variables are influenced by details such as the probability density estimation, discretization rules, and numerical integration necessitates careful method design to ensure the robustness of information-based rankings.

The Sharma–Mittal entropy provides a broader framework of generalized entropy related to the Rényi and Tsallis families, thanks to its two-parameter structure [16,17,18]. However, fixing the alpha and beta parameters at a single point in applied parameter selection studies may lead to the metric becoming dependent on parameter selection. The central idea of this study is to evaluate the conditional entropy surface and the information gain surface volumetrically across the entire selected alpha–beta region, rather than selecting a single parameter pair. Thus, the variable importance is defined not through local behavior at a specific parameter setting, but through the holistic structure of entropic behavior in the parameter space [19,20,21,22].

The original contribution of this study stems from the combination of two components: a density estimation without discretization for the continuous variables and the evaluation of volumetric entropy over the parameter space. In the first component, the interval-based discretization commonly used in entropy calculations is completely abandoned; the marginal density of the target, the joint density of the target and the candidate variable, and the conditional density derived from them are obtained using a Gaussian kernel density estimation. Instead of being user-defined, the bandwidth is automatically selected using Scott’s rule, ensuring that all datasets and all variables are subjected to the same computational protocol. As a critical design decision, the bandwidth in the direction of the target is kept constant in both the marginal and joint density estimations; this ensures that marginalizing the joint density over the explanatory variable fully restores the marginal density and structurally prevents the conditional entropy from artificially exceeding the marginal entropy. In addition, the conditional density generated via partitioning is renormalized at each conditioning point to satisfy the unit integral condition; this correction is necessary because the (1 − *β*)/(1 − *α*) exponent in the entropy expression amplifies normalization errors. Together, these two measures eliminate the problem of negative information gain observed in the continuous variables and ensure the production of positive scores across all six datasets.

The second component involves decoupling the entropy metric from parameter selection. Due to its two-parameter structure, the Sharma–Mittal entropy encompasses the Rényi, Tsallis, and Shannon metrics as special cases; however, in practice, fixing *α* and *β* at a single point causes the derived variable importance to become dependent on this arbitrary choice. In the proposed framework, the conditional entropy and information gain are treated not as values at a specific parameter pair, but as surfaces defined across the entire region Ω = [0.05, 0.95]^2^, and the volumes beneath these surfaces are calculated using two-dimensional numerical integration. Thus, variable importance is defined not by the local behavior at a single point in parameter space, but by the holistic structure of entropic behavior. The volume components correspond to three different interpretations: the conditional entropy volume (PICSME) represents the absolute magnitude of the residual uncertainty regarding the target; the gain volume (PIGSME) represents the total reduction in uncertainty; and the normalized gain (NIGSME) represents the ratio of this reduction to the target’s total entropy volume (see Appendix A). Since the latter is dimensionless and upper bounded, it is suitable for comparisons across datasets. This combination of non-decomposed density estimation with volumetric parameter integration constitutes, to the best of our knowledge, the first parameter-independent Sharma–Mittal feature selection criterion proposed for continuous variables.

## 2. Materials and Methods

### 2.1. Sharma–Mittal Entropy and Its Representation via Rényi Entropy

**Definition 1** (Continuous Sharma–Mittal entropy)**.** *For* α>0 *,* α≠1, and β≠1 *, the continuous Sharma–Mittal entropy is defined as*

(1) $$H_{\alpha ,\beta }\left(Y\right)=\frac{{\left[\int f_{Y}{\left(y\right)}^{a}dy\right]}^{\frac{1-\beta }{1-\alpha }}-1}{1-\beta }$$

*To simplify the derivation, let us define the Rényi differential entropy of order α as*

(2) $$H_{\alpha }^{R}\left(Y\right)=\frac{1}{1-a}ln\int f_{Y}{\left(y\right)}^{\alpha }dy\Leftrightarrow \int f_{Y}^{\alpha }dy=e^{\left(1-\alpha \right)H_{\alpha }^{R}\left(Y\right)}$$

**Lemma 1** (Representation of Sharma–Mittal entropy via Rényi entropy)**.** *Substituting Equation (2) into Equation (1) yields*

(3) $$H_{\alpha ,\beta }\left(Y\right)=\frac{e^{\left(1-\beta \right)H_{\alpha }^{R}\left(Y\right)}-1}{1-\beta }=\varphi_{\beta }\left(H_{\alpha }^{R}\left(Y\right)\right),\;\;\varphi_{\beta }\left(t\right)=\frac{e^{(1-\beta )t}-1}{1-\beta }$$

*The transformation* $\varphi_{\beta }$ *has the following properties:*

1.
$\varphi_{\beta }^{\prime }\left(t\right)=e^{\left(1-\beta \right)t}>0$ *: Is definitely increasing;*
2.
$\varphi_{\beta }^{\prime \prime }\left(t\right)=\left(1-\beta \right)e^{\left(1-\beta \right)t}$ *: Is definitely convex for* β<1 *;*
3.
$\underset{\beta \to 1}{lim}\varphi_{\beta }\left(t\right)=t$ *: Is reduced to linear.*

**Proof of Lemma** **1.** The representation follows directly by substitution. The derivative properties follow immediately from the definition of $\varphi_{\beta }\left(t\right)$. For the limiting case, letting δ=1−β→0 and using the standard limit $\frac{e^{\delta t}-1}{\delta }\to t$ gives the stated result. □

Note 1: Under Appendix B Assumption 2, all values of *β* considered in this study satisfy β < 1. Consequently, $\varphi_{\beta }$ is strictly convex over the entire parameter integration domain. This property will be particularly relevant to the discussion in Section 2.4.3.

**Proposition 1** (Special cases)**.** *The following reductions follow directly from the representation in Equation (3):*

(4) $$\beta \to 1:\;H_{\alpha ,\beta }\left(Y\right)\to H_{\alpha }^{\beta }\left(Y\right),\;\;(Rényi\;Entropy)$$

(5) $$\beta =\alpha :\;H_{\alpha ,\beta }\left(Y\right)=\frac{\left(\int f_{y}^{a}\right)-1}{1-\alpha },\;\;(Tsallis\;Entropy)$$

(6) $$\alpha \to 1,\;\beta \to 1:\;H_{\alpha ,\beta }\left(Y\right)\to \;h\left(Y\right)=-\int f_{Y}lnf_{Y},\;\;\;(Shannon\;differential\;entropy)$$

**Figure 1:**
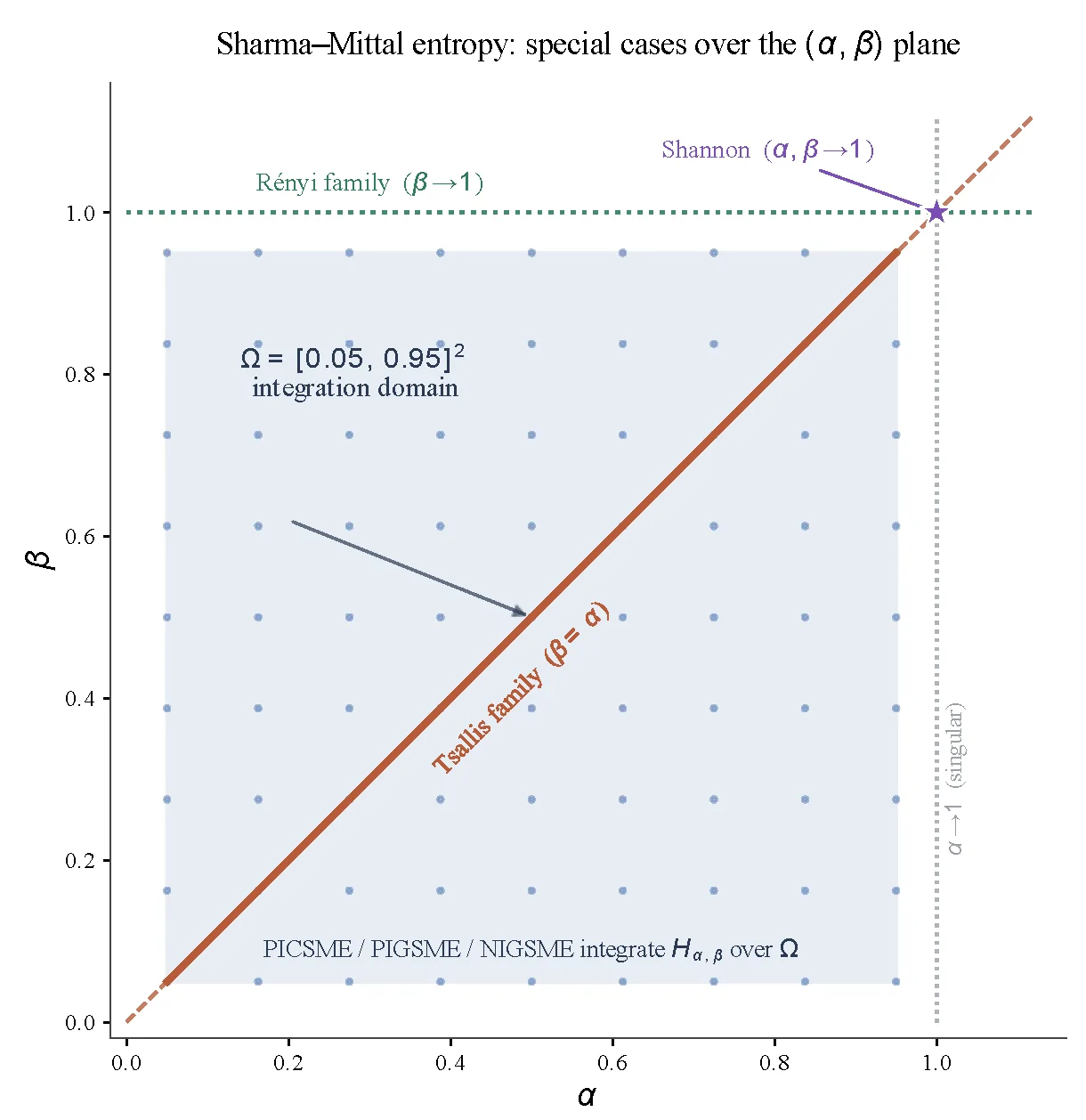
The location of the Sharma–Mittal entropy in the alpha–beta parameter plane and the integration region Ω = [0.05; 0.95] × [0.05; 0.95] used in this study.

*These three reductions demonstrate the unifying nature of the Sharma–Mittal entropy family and are consistent with the parameter space relationships illustrated in Figure 1.*

**Proof of Proposition** **1.** The representation follows directly by substitution. The derivative properties Equation (4) follow from property (iii) of Lemma 1. Equation (5) is obtained by setting *β* = *α*, for which (1 − *β*)/(1 − *α*) = 1. For Equation (6), first taking *β* → 1 yields the Rényi entropy; subsequently, taking *α* → 1 and applying L’Hôpital’s rule gives $\underset{\alpha \to 1}{lim}\frac{1}{1-\alpha }ln\int f_{y}^{a}=\;-\int f_{Y}lnf_{Y}$. Thus, the Shannon differential entropy is recovered. □

### 2.2. Conditional Entropy and Kernel Density Estimation

#### 2.2.1. Conditional Sharma–Mittal Entropy

**Definition 2** (Conditional Sharma–Mittal entropy)**.** *The conditional Sharma–Mittal entropy is defined as*

(7) $$H_{\alpha ,\beta }\left(Y|X_{j}\right)=\int f_{X_{j}}\left(x\right)\left\{\frac{{\left[\int {\left(\frac{f_{Y,X_{j}}\left(y,x\right)}{f_{X_{j}}\left(x\right)}\right)}^{\alpha }dy\right]}^{\frac{1-\beta }{1-\alpha }}-1}{1-\beta }\right\}dx=E_{X_{j}}\left[\varphi_{\beta }\left(H_{\alpha }^{R}\left(Y|X_{j}\right)\right)\right]\;$$

*Thus, the conditional index is obtained by applying the transformation *$\varphi_{\beta }$* to the pointwise conditional Rényi entropy evaluated at each value of x, followed by averaging with respect to the marginal distribution of *$x_{j}$.

#### 2.2.2. Kernel Density Estimation Framework

Since the density functions appearing in Equation (7) are unknown in practice, they are estimated using a Gaussian kernel density estimation (KDE). For the observations ${\left\{\left(y_{i},x_{ij}\right)\right\}}_{i=1}^{n}$, the required density estimates are given by

(8) $${\hat{f}}_{Y}\left(y\right)=\frac{1}{nh_{Y}}\sum_{i=1}^{n}K\left(\frac{y-y_{i}}{h_{Y}}\right),\;\;\;\;\;\;\;K\left(u\right)=\frac{1}{\sqrt{2\pi }}e^{-u^{2}/2},\;$$

(9) $${\hat{f}}_{Y,X_{j}}\left(y,x\right)=\frac{1}{nh_{Y}h_{X}}\sum_{i=1}^{n}K\left(\frac{y-y_{i}}{h_{Y}}\right)K\left(\frac{x-x_{ij}}{h_{X}}\right),$$

(10) $${\hat{f}}_{X_{j}}\left(x\right)=\int {\hat{f}}_{Y,X_{j}}\left(y,x\right)\;dy=\frac{1}{nh_{X}}\sum_{i=1}^{n}K\left(\frac{x-x_{ij}}{h_{X}}\right),$$

(11) $${\hat{f}}_{Y|X_{j}}\left(y|x\right)=\frac{{\hat{f}}_{Y,X_{j}}\left(y,x\right)}{{\hat{f}}_{X_{j}}\left(x\right)}.$$

The bandwidths are automatically determined using Scott’s rule,

(12) $$h=\hat{\sigma }\;n^{-1/\left(d+4\right)},$$

where d denotes the dimensionality of the density estimate (d = 1 for marginal density estimation and d = 2 for joint density estimation), and $\hat{\sigma }$ denotes the sample standard deviation of the corresponding variable.

**Proposition 2** (Bandwidth consistency)**.** *If the bandwidth in the y-direction in Equation (9) is chosen to be identical to that used in Equation (8), marginalizing the joint density estimator over x exactly recovers the marginal density estimator:*

(13) $$\int {\hat{f}}_{Y,X_{j}}\left(y,x\right)\;dx={\hat{f}}_{Y}\left(y\right),\;\;\mathrm{for}\;\mathrm{all}\;y.$$

**Proof of Proposition** **2.** Integrating Equation (9) with respect to *x* gives

$$\int {\hat{f}}_{Y,X_{j}}\left(y,x\right)\;dx=\frac{1}{nh_{Y}}\sum_{i=1}^{n}K\left(\frac{y-y_{i}}{h_{Y}}\right)\underset{=1}{\underbrace{\frac{1}{h_{X}}\int K\left(\frac{x-x_{ij}}{h_{X}}\right)dx}}={\hat{f}}_{Y}\left(y\right),$$

since the Gaussian kernel integrates to one. Therefore, the equality holds provided that the same bandwidth in the *y*-direction is used in both density estimators. □

Note 2 (Methodological consideration): Scott’s rule in Equation (12) yields $h\alpha n^{-\frac{1}{5}}$ for a marginal density estimator and $h\alpha n^{-\frac{1}{6}}$ for a two-dimensional joint density estimator. If these bandwidths are selected independently, the bandwidth in the *y*-direction generally differs between the marginal and joint density estimators, thereby violating the exact equality in Equation (13). Consequently, the estimated conditional density may become more dispersed than the corresponding marginal density, potentially leading to negative values of the estimated conditional Rényi entropy $H_{\alpha }^{R}\left(Y|X_{j}\right)$ and, hence, negative information gain values. To prevent this inconsistency, the bandwidth in the *y*-direction is kept identical in both estimators throughout this study, thereby ensuring the consistency property established in Proposition 2.

**Proposition 3** (Conditional renormalization)**.** *The conditional density constructed via (11) is not generally guaranteed to integrate to one. For each x, define the renormalized conditional density*

(14) $${\tilde{f}}_{Y|X_{j}}\left(y|x\right)=\frac{{\hat{f}}_{Y|X_{j}}\left(y|x\right)}{\int {\hat{f}}_{Y|X_{j}}\left(u|x\right)\;du}$$

*so that* $\int {\tilde{f}}_{Y|X_{j}}\left(y|x\right)\;dy=1$ *holds by construction.*

**Proof of Proposition** **3.** We have $\int {\hat{f}}_{Y|X_{j}}\left(y|x\right)\;dy={\hat{f}}_{X_{j}}^{\left(joint\right)}\left(x\right)/{\hat{f}}_{X_{j}}\left(x\right)$. Since the numerator and denominator arise from independent smoothing procedures, this ratio may deviate from one. In Equation (14), the denominator is set exactly equal to this ratio, so the integral equals one after division. The effect of this deviation on the entropy is amplified by the exponent (1 − *β*)/(1 − *α*); normalization therefore cannot be omitted. □

#### 2.2.3. Numerical Integration

Integrals of the form $\int f^{\alpha }$ are computed via the trapezoidal rule on a uniform grid with $N_{y}=N_{x}=256$ points. With weights $w_{1}=w_{N}=\Delta /2\;and\;w_{i}=\Delta \left(2\leq i\leq N-1\right)$,

(15) $$\int f{\left(y\right)}^{\alpha }\;dy\approx \sum_{i=1}^{N_{y}}w_{i}f{\left(y_{i}\right)}^{\alpha }.$$

The grid boundaries are determined by extending the sampling range by 4 *h* in each direction, thereby bounding the mass of the Gaussian kernel’s tail lying outside the grid to $\Phi \left(-4\right)\approx 3.17\times 10^{-5}$.

**Proposition 4** (Convergence of the trapezoidal rule)**.** *Under Assumption 1,* $g\left(y\right)=f{\left(y\right)}^{a}$ *is twice continuously differentiable, and the error of the trapezoidal rule is of order*

(16) $$\left|\int_{a}^{b}g\left(y\right)\;dy-\sum_{i}w_{i}g\left(y_{i}\right)\right|\leq \frac{\left(b-a\right)\Delta^{2}}{12}\underset{y\in \left[a,b\right]}{\max}\left|g^{\prime \prime }\left(y\right)\right|=O\left(N^{-2}\right)$$

*Consequently, for* N=256 *the numerical error is negligible relative to the KDE’s own estimation error.*

### 2.3. Derivation of the Three Indices

The three indices defined in this section rely on the volumes enclosed by the surfaces ${\hat{H}}_{\alpha ,\beta }(Y)$ and ${\hat{H}}_{\alpha ,\beta }\left(Y|X_{j}\right)$ over the integration region $D=\left[\alpha_{Lower},\alpha_{Upper}\right]\times \left[\beta_{Lower},\beta_{Upper}\right]={\left[0.05,0.95\right]}^{2}$ (see Appendix B). The location of the Sharma–Mittal entropy in the (*α*, *β*) plane, together with the integration region Ω used throughout this study, is shown in Figure 1. The Rényi family is obtained for the limit β→1; the Tsallis family lies on the line *β* = *α*; and the Shannon entropy is recovered at the intersection of the two families, (α,β)→(1,1).

The surfaces themselves are obtained, for each candidate $X_{j}$, from the Gaussian kernel density estimates [23] of the target’s marginal density ${\hat{f}}_{Y}$, the joint density ${\hat{f}}_{Y,X_{j}}$, and the conditional density ${\hat{f}}_{Y|X_{j}}$. Rather than being set manually across datasets, the bandwidth is determined using Scott’s rule [24]. For each candidate variable, the marginal density of the target and the bivariate joint density are estimated, and the conditional density is derived from these (see Equation (17)). Applying the same estimation procedure uniformly across all datasets and all variables is intended to ensure that differences in the resulting scores stem, as far as possible, from the variable–target relationship rather than from methodological artefacts [25,26,27]. The resulting surfaces are constructed over a fixed 60×60(α,β) grid (see Appendix C).

#### 2.3.1. PICSME (Parameter-Integrated Conditional Sharma–Mittal Entropy): Conditional Uncertainty Volume

Rather than considering a single fixed pair (*α*,*β*), we account for the entire region *D*:

(17) $${Ω}_{j}^{CE}=\left\{\left(\alpha ,\beta ,z\right):\left(\alpha ,\beta \right)\in D,\;0\leq z\leq {\hat{H}}_{\alpha ,\beta }\left(Y|X_{j}\right)\right\},\;$$

(18) $$V_{j}^{CE}={∭}_{\Omega_{j}^{CE}}1\;dV=\int_{\alpha_{Lower}}^{\alpha_{Upper}}\int_{\beta_{Lower}}^{\beta_{Upper}}{\hat{H}}_{\alpha ,\beta }\left(Y|X_{j}\right)\;d\beta \;d\alpha .$$

Since the inner integral over *z* directly returns the surface height, the triple integral reduces to a double integral. Dividing by the area of the region yields a scale-free index:

(19) $${\mathrm{PICSME}}_{j}=\frac{V_{j}^{CE}}{\left|D\right|}=\frac{1}{\left|D\right|}\int_{\alpha_{Lower}}^{\alpha_{Upper}}\int_{\beta_{Lower}}^{\beta_{Upper}}{\hat{H}}_{\alpha ,\beta }\left(Y|X_{j}\right)\;d\beta \;d\alpha$$

Note 3: In (19), ${\hat{H}}_{\alpha ,\beta }\left(Y|X_{j}\right)$ is the average value over *D*. A low PICSME means that the residual uncertainty about Y, once $X_{j}$ is observed, is small; it is therefore used in ascending order in the ranking.

**Figure 2:**
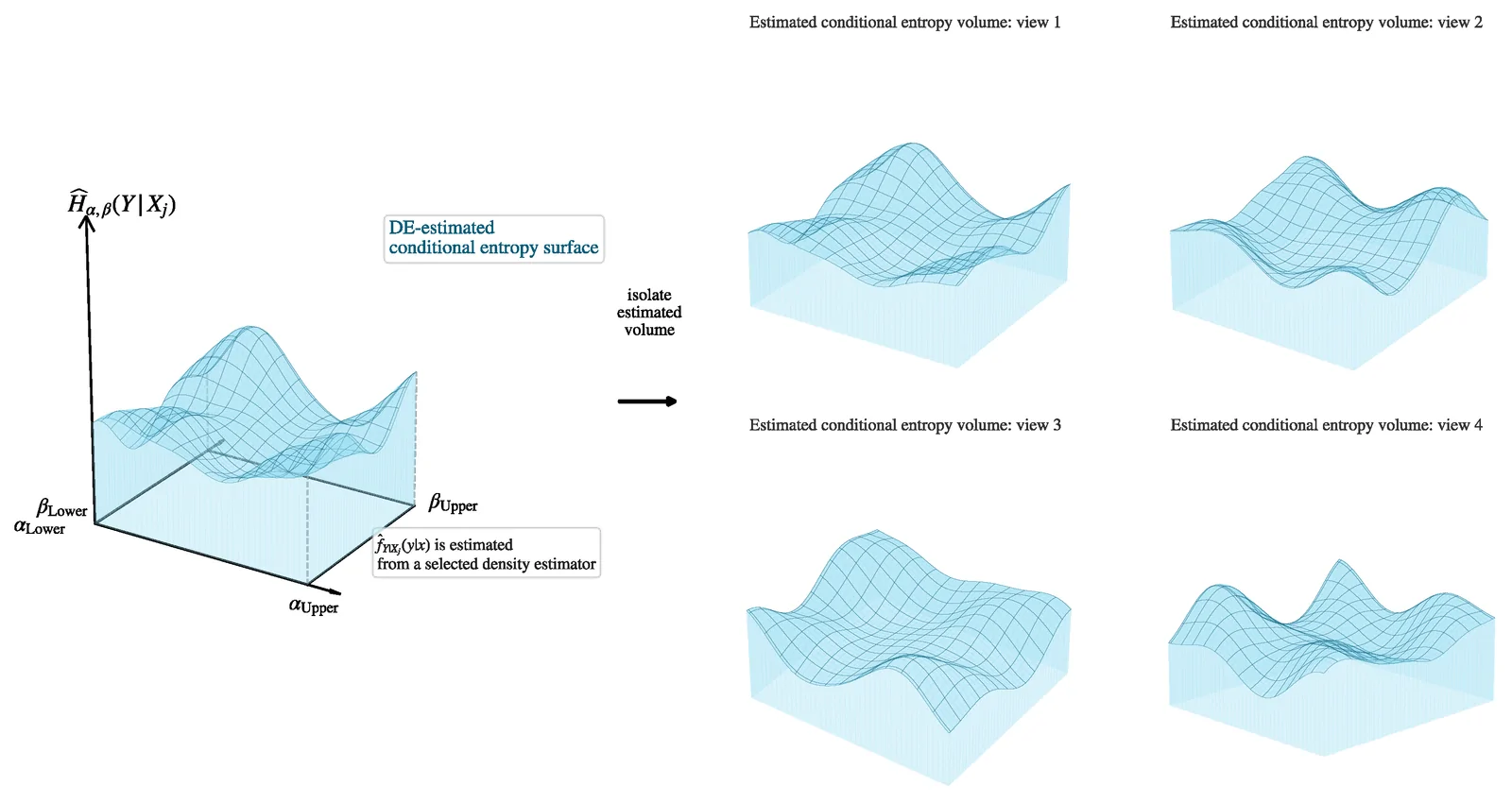
Parameter-integrated conditional Sharma–Mittal entropy: Volumetric interpretation of the conditional entropy surface in the alpha–beta plane, obtained using the selected density estimation approach.

A volumetric representation of this conditional entropy surface, obtained using the selected density estimation approach, is shown in Figure 2.

#### 2.3.2. PIGSME (Parameter-Integrated Gain of Sharma–Mittal Entropy): Information Gain Volume

The volume of the difference between the target’s marginal entropy surface and the conditional entropy surface is given by

(20) $$V_{j}^{IG}=\int_{\alpha_{Lower}}^{\alpha_{Upper}}\int_{\beta_{Lower}}^{\beta_{Upper}}\left[{\hat{H}}_{\alpha ,\beta }\left(Y\right)-{\hat{H}}_{\alpha ,\beta }\left(Y|X_{j}\right)\right]d\beta \;d\alpha ,$$

(21) $${PIGSME}_{j}=\frac{V_{j}^{IG}}{\left|D\right|}=\frac{1}{\left|D\right|}\int_{\alpha_{Lower}}^{\alpha_{Upper}}\int_{\beta_{Lower}}^{\beta_{Upper}}\left[{\hat{H}}_{\alpha ,\beta }\left(Y\right)-{\hat{H}}_{\alpha ,\beta }\left(Y|X_{j}\right)\right]d\beta d\alpha$$

Denoting the target’s marginal entropy volume by

(22) $$V_{Y}=\int_{\alpha_{Lower}}^{\alpha_{Upper}}\int_{\beta_{Lower}}^{\beta_{Upper}}{\hat{H}}_{\alpha ,\beta }\left(Y\right)\;d\beta \;d\alpha$$

linearity of the integral gives

(23) $${PIGSME}_{j}=\frac{V_{Y}}{\left|D\right|}-{PICSME}_{j}$$

This relation is used in Section 2.5.

**Figure 3:**
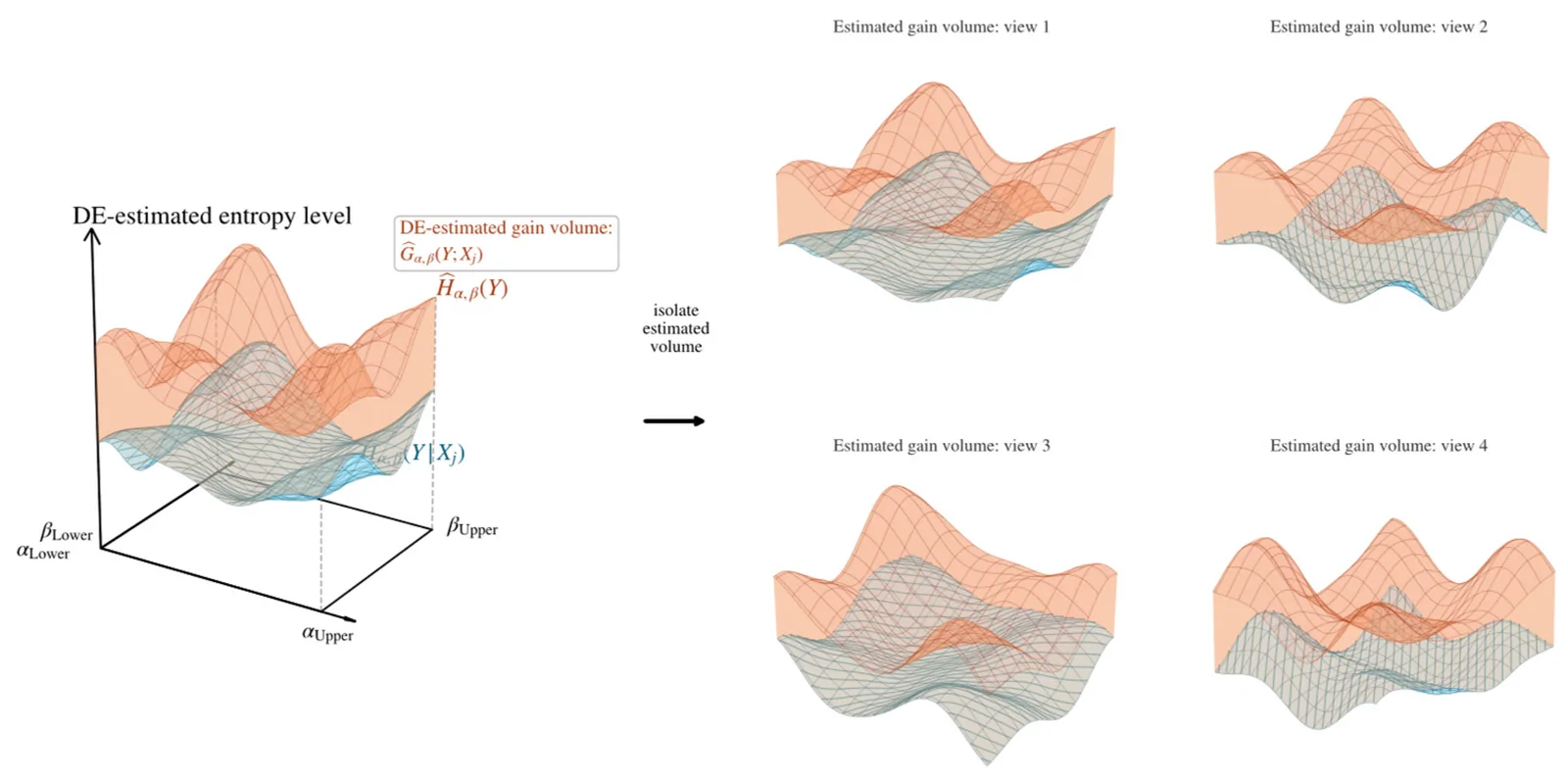
Parameter-integrated Sharma–Mittal entropy information gain: A conceptual illustration of the volume of the difference between the marginal and conditional entropy surfaces.

A conceptual illustration of this difference in volume between the marginal and conditional entropy surfaces is presented in Figure 3.

#### 2.3.3. NIGSME (Normalized Integrated Gain of Sharma–Mittal Entropy): Normalized Information Gain

To reduce the scale effects across the different datasets, the gain volume is divided by the target’s total entropy volume:

(24) $${\mathrm{NIGSME}}_{j}=\frac{V_{j}^{IG}}{V_{Y}}=\frac{\int_{\alpha_{Lower}}^{\alpha_{Upper}}\int_{\beta_{Lower}}^{\beta_{Upper}}\left[{\hat{H}}_{\alpha ,\beta }\left(Y\right)-{\hat{H}}_{\alpha ,\beta }\left(Y|X_{j}\right)\right]d\beta \;d\alpha }{\int_{\alpha_{Lower}}^{\alpha_{Upper}}\int_{\beta_{Lower}}^{\beta_{Upper}}{\hat{H}}_{\alpha ,\beta }\left(Y\right)\;d\beta \;d\alpha }$$

Decomposing the numerator integral yields an equivalent expression:

(25) $${\mathrm{NIGSME}}_{j}=\frac{V_{Y}-\iint {\hat{H}}_{\alpha ,\beta }\left(Y|X_{j}\right)\;d\beta \;d\alpha }{V_{Y}}=1-\frac{\int_{\alpha_{Lower}}^{\alpha_{Upper}}\int_{\beta_{Lower}}^{\beta_{Upper}}{\hat{H}}_{\alpha ,\beta }\left(Y|X_{j}\right)\;d\beta \;d\alpha }{\int_{\alpha_{Lower}}^{\alpha_{Upper}}\int_{\beta_{Lower}}^{Upper}{\hat{H}}_{\alpha ,\beta }\left(Y\right)\;d\beta \;d\alpha }=1-\frac{\left|D\right|\cdot {\mathrm{PICSME}}_{j}}{V_{Y}}$$

Equation (25) allows for the NIGSME to be interpreted as “the proportional share of the uncertainty in the target that is eliminated by $X_{j}$.”

**Figure 4:**
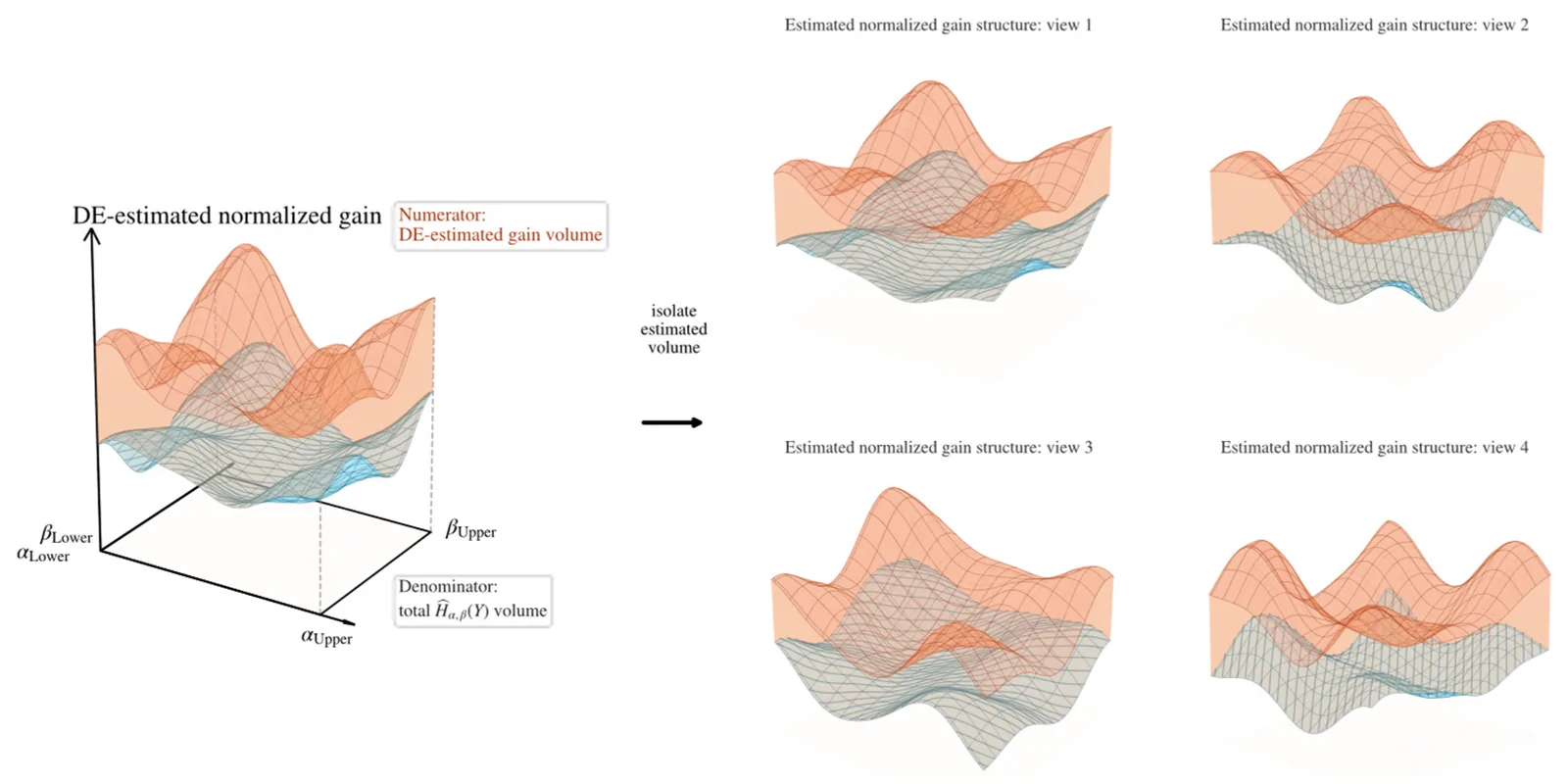
Normalized parameter-integrated Sharma–Mittal entropy information gain: Normalization of the information gain volume relative to the total Sharma–Mittal entropy volume of the target.

The representation of the normalized gain volume as the ratio of the gain volume to the target’s total Sharma–Mittal entropy volume is shown in Figure 4.

All three indices are calculated by numerically integrating the surfaces over *D*, following Equations (19), (21), and (24); the entire calculation process is detailed in Appendix C.

### 2.4. Basic Properties and Proofs

#### 2.4.1. Behavior Under Independence

**Theorem 1** (Exact zero under independence)**.** *If* $X_{j}$ *and Y are independent, then for every* (α,β) ϵ D *,* $H_{\alpha ,\beta }\left(Y|X_{j}\right)=H_{\alpha ,\beta }\left(Y\right),\;$ *and, consequently,*

(26) $${PIGSME}_{j}=0,\;\;{NIGSME}_{j}=0,\;\;{PICSME}_{j}=\frac{V_{Y}}{\left|D\right|}.$$

**Proof of Theorem** **1.** By independence, $f_{Y|X_{j}}\left(\cdot |x\right)=f_{Y}\left(\cdot \right)$ for every *x*. Hence, $H_{\alpha }^{R}\left(Y|X_{j}=x\right)$ is a constant independent of *x*, equal to $H_{\alpha }^{R}\left(Y\right)$. Taking the expectation in (7), the expected value of a constant returns the constant itself: $H_{\alpha ,\beta }\left(Y|X_{j}\right)=E_{X_{j}}\;\left[\varphi_{\beta }\left(H_{\alpha }^{R}\left(Y\right)\right)\right]=\varphi_{\beta }\;\left(H_{\alpha }^{R}\left(Y\right)\right)=H_{\alpha ,\beta }\left(Y\right).$ Since the difference is zero at every point, the integral in (20) is zero; (26) then follows. □

Note 4: Theorem 1 is important: the proposed criterion yields exactly zero in the case of independence. Negative values therefore stem not from independence, but from structural causes discussed in the next section.

#### 2.4.2. Shannon Limit and Classical Information Gain

**Theorem 2** (Non-negativity at the Shannon corner)**.** *As (α,β) → (1,1),*

(27) $$H_{\alpha ,\beta }\left(Y\right)-H_{\alpha ,\beta }\left(Y|X_{j}\right)\;⟶\;h\left(Y\right)-h\left(Y|X_{j}\right)=I\left(Y;X_{j}\right)\geq 0.$$

**Proof of Theorem** **2.** By Proposition 1, $H_{\alpha ,\beta }\left(Y\right)\to h\left(Y\right)$. For the conditional term, by Lemma 1(3), $\varphi_{\beta }$ converges to the identity, and $E_{X_{j}}\left[h\left(Y|X_{j}=x\right)\right]=h\left(Y|X_{j}\right)$. The difference is then the classical mutual information, which, being an instance of the Kullback–Leibler divergence, is non-negative. □

#### 2.4.3. Sign Decomposition in the General Parameter Region

Outside the Shannon corner, non-negativity is not automatic. The following decomposition identifies the exact cause.

**Theorem 3** (Sign decomposition)**.** *Let* $m_{j}:=E_{X_{j}}\;\left[H_{\alpha }^{R}\left(Y|X_{j}\right)\right]$ *and let the Jensen gap be*

(28) $$J_{\alpha ,\beta }\left(X_{j}\right):=E_{X_{j}}\;\left[\varphi_{\beta }\;\left(H_{\alpha }^{R}\left(Y|X_{j}\right)\right)\right]-\varphi_{\beta }\left(m_{j}\right)\geq 0$$

*Then, the pointwise gain*

(29) $$G_{\alpha ,\beta }\left(X_{j}\right):=H_{\alpha ,\beta }\left(Y\right)-H_{\alpha ,\beta }\left(Y|X_{j}\right)=\underset{A_{\alpha ,\beta }\left(X_{j}\right)}{\underbrace{\left[\varphi_{\beta }\;\left(H_{\alpha }^{R}\left(Y\right)\right)-\varphi_{\beta }\left(m_{j}\right)\right]}}-J_{\alpha ,\beta }\left(X_{j}\right)$$

*decomposes as above, and*

(30) $$G_{\alpha ,\beta }\left(X_{j}\right)\geq 0⟺A_{\alpha ,\beta }\left(X_{j}\right)\geq J_{\alpha ,\beta }\left(X_{j}\right)$$

**Proof of Theorem** **3.** Using (7) and (3), we write $G_{\alpha ,\beta }=\varphi_{\beta }\;\left(H_{\alpha }^{R}\left(Y\right)\right)-E_{X_{j}}\;\left[\varphi_{\beta }\left(H_{\alpha }^{R}\left(Y|X_{j}\right)\right)\right]$. Adding and subtracting $\pm \varphi_{\beta }\left(m_{j}\right)$ on the right-hand side directly yields (29). That Jα,β ≥0 follow from the convexity of $\varphi_{\beta }$ for *β* < 1 together with Jensen’s inequality (Lemma 1(2)). Since $\varphi_{\beta }$ is strictly increasing, ${\mathrm{sign}A}_{\alpha ,\beta }=\mathrm{sign}\;\left(H_{\alpha }^{R}\left(Y\right)-m_{j}\right)$. □

**Corollary 1** (Magnitude of the Jensen gap)**.** *By the second-order Taylor expansion of* $\varphi_{\beta }$ *,*

(31) $$J_{\alpha ,\beta }\left(X_{j}\right)\approx \frac{1}{2}{\varphi_{\beta }}^{\prime \prime }\left(m_{j}\right)\;{Var}_{X_{j}}\;\left[H_{\alpha }^{R}\left(Y|X_{j}\right)\right]=\frac{1}{2}\left(1-\beta \right)e^{\left(1-\beta \right)m_{j}}\;{Var}_{X_{j}}\;\left[H_{\alpha }^{R}\left(Y|X_{j}\right)\right]$$

That is, the negative pressure grows together with (1 − *β*) and the variance of the conditional Rényi entropy across *x*.

**Corollary 2** (Condition for negativity to arise)**.** *Equations (30) and (31) together show that the gain can turn negative when a variable is a weak informer (small* $A_{\alpha ,\beta }$ *) and its conditional entropy is dispersed across x (large Var). However, once the corrections in Note 2 and Proposition 3 are applied,* ${PIGSME}_{j}>0\;\mathrm{and}\;{NIGSME}_{j}>0$ *are obtained for all variables across the entire set of six datasets.*

#### 2.4.4. Boundedness

**Proposition 5** (Upper bound of NIGSME)**.** *As long as* ${\hat{H}}_{\alpha ,\beta }\left(Y|X_{j}\right)\geq 0$ *,*

(32) $${NIGSME}_{j}\leq 1,$$

*and under the condition* $G_{\alpha ,\beta }\geq 0,\;0\leq {NIGSME}_{j}\leq 1$ *.*

**Proof of Proposition** **5.** From (23), ${NIGSME}_{j}=1-\left|D\right|\;{PICSME}_{j}/V_{Y}$; since ${PICSME}_{j}\geq 0$, the second term is non-negative, and so the expression does not exceed 1. The lower bound follows from the non-negativity condition of Theorem 3. □

### 2.5. The Order Equivalence of the Three Indices

**Theorem 4** (Order equivalence)**.** *In a fixed data set and a fixed region D,* $V_{y}$ *is a constant independent of the candidate variable index. In this case,*

(33) $${PIGSME}_{j}=C_{1}-{PICSME}_{j},\;\;C_{1}=\frac{V_{Y}}{\left|D\right|}>0,$$

(34) $${NIGSME}_{j}=1-\frac{\left|D\right|}{V_{Y}}\;{PICSME}_{j}=\frac{\left|D\right|}{V_{Y}}\;{PIGSME}_{j},$$

*and both indices are exact decreasing affine transformations of* ${PICSME}_{j}$ *. Therefore,*

(35) $${PICSME}_{i}<{PICSME}_{j}⟺{PIGSME}_{i}>{PIGSME}_{j}⟺{NIGSME}_{i}>{NIGSME}_{j},$$

*In other words, an increasing ranking according to PICSME is equivalent to a decreasing ranking according to PIGSME and NIGSME.*

**Proof of Theorem** **4.** $V_{y}$ depends solely on the target’s marginal entropy surface; it has no relation to the candidate variable and therefore remains constant for all j in the dataset. Equation (23) directly yields (33), and (34) follows from (25). Since in both transformations the coefficient of ${PICSME}_{j}$ is, respectively, −1 and $-\left|D\right|/V_{Y}<0$, the transformations are strictly decreasing; a strictly monotone transformation reverses the ordering but preserves the relative order, which gives (35).

Note 5 (Interpretation): Theorem 4 shows that the three indices producing the same ordering across all datasets in the main text is not an empirical observation but a mathematical necessity. The three indices should therefore be interpreted not as three independent selection criteria, but as three different reporting scales of a single ranking criterion:

- PICSME: Absolute conditional uncertainty volume (has units; lower is better);
- PIGSME: Absolute gain volume (has units; higher is better);
- NIGSME: Relative gain (unitless; ≤1; suitable for cross-dataset comparison).

For the three scales to be able to produce distinct orderings, additional extensions are required, such as standardization across datasets or the use of different objective functions. □

### 2.6. Datasets and Preprocessing

The experimental evaluation was conducted on six regression datasets that differed in terms of sample size, the number of explanatory variables, and the application domain. Airfoil Self-Noise represents an acoustic/aerodynamic context [28,29], AirQualityUCI represents air quality [30,31], BodyFat represents body composition [32], Meteorology represents hydrometeorology [33,34,35], Concrete represents materials engineering [36,37], and WineQualityWhite represents food/wine quality [38,39]. This diversity ensured that the proposed indices could be evaluated within an experimental framework suitable for examining similarities and differences between methods in both low-dimensional and high-dimensional physical, sensor-based, and compositional data generation processes. The sample sizes of the datasets, the number of explanatory variables used, the target variables, and the data processing steps are summarized in Table 1.

**Figure 5:**
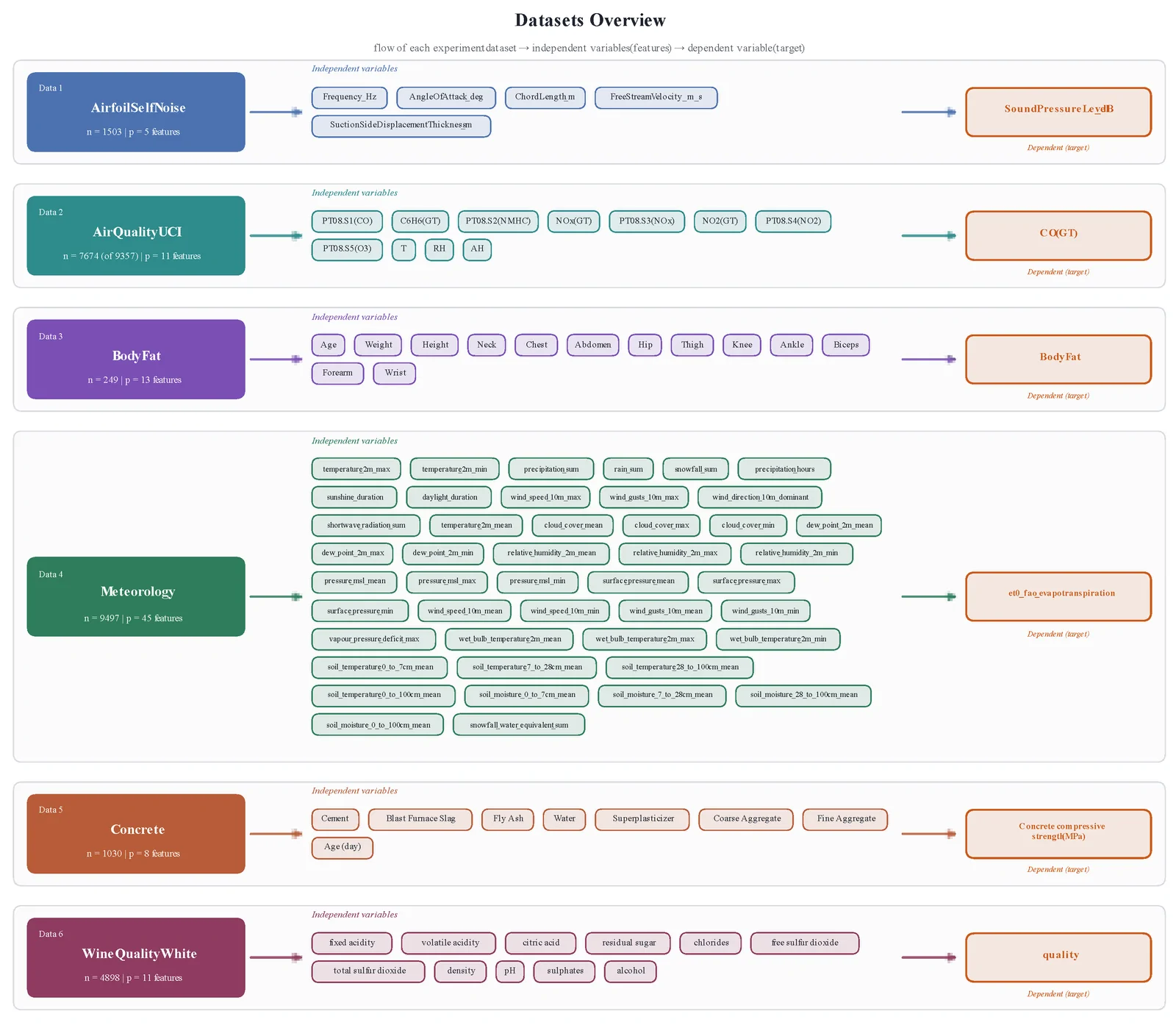
Experimental flow of the six datasets: the relationship between the dataset, the independent variables, and the dependent target variable.

The analytical structure between the independent variables and the target variable used in each dataset is shown in Figure 5. In the Airfoil Self-Noise dataset, the variable names were defined manually because the source file lacked a header row; in the AirQualityUCI dataset, invalid target observations, along with the date, time, and NMHC(GT) columns, were excluded. In the Meteorology dataset, the date, sunrise, sunset, and weather_code variables were excluded from the analysis; in the AirQualityUCI and Meteorology datasets, a deterministic subsampling of 5000 observations was applied to limit the cost of two-variable density estimation. In the other datasets, all available observations and continuous explanatory variables were directly included in the analysis.

## 3. Results

### 3.1. Data 1—Airfoil Self-Noise Dataset

**Figure 6:**
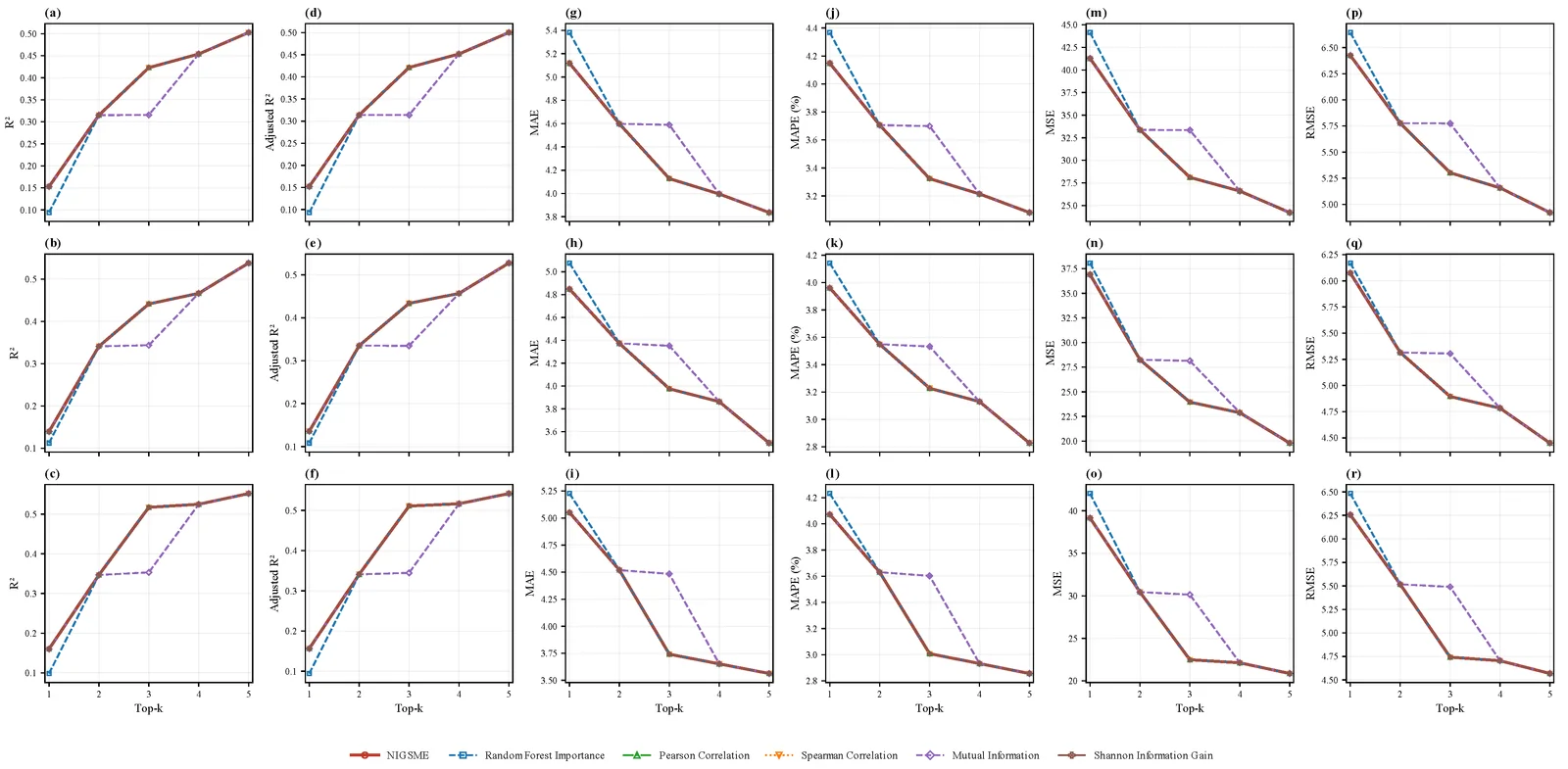
OLS estimation performance of NIGSME and comparison methods on top-k feature subsets in the Airfoil Self-Noise dataset. (Note: Rows correspond to partitions and columns to performance metrics; panel letters progress from top to bottom across each column. Top row (**a**,**d**,**g**,**j**,**m**,**p**)—training partition; middle row (**b**,**e**,**h**,**k**,**n**,**q**)—test partition; bottom row (**c**,**f**,**i**,**l**,**o**,**r**)—validation set. The columns, from left to right, contain the R^2^, adjusted R^2^, MAE, MAPE, MSE, and RMSE metrics, respectively. In each panel, the horizontal axis represents the number of selected features (top-k), and the vertical axis represents the corresponding performance metric. Feature rankings are calculated only for the training set).

Figure 6 presents the performance of OLS models built using the top-k subsets derived from the six feature selection methods on the Airfoil Self-Noise dataset, showing their performance on the training (69.99%, *n* = 1052), test (%14.97, *n* = 225), and validation (%15.04, *n* = 226) partitions; the rows correspond to the training, test, and validation partitions, respectively, while the columns correspond to the R^2^, adjusted R^2^, MAE, MAPE, MSE, and RMSE metrics. A common structural feature of the panels is that all method curves overlap at k = 2, k = 4, and k = 5 (Figure 6a–r). This is not a coincidence but a direct result of the rankings: at the mentioned k values, the subsets selected by the methods overlap as a whole (see Table 2), even if the positions of the variables within the ranks differ; since the OLS estimate depends solely on the composition of the subset, the same model is obtained. Since all methods converge to the full model at k = 5, this point is, by definition, not discriminative. Accordingly, a distinction between the methods is observed at only two points: k = 1, where random forest importance places SuctionSideDisplacementThickness_m in the first position instead of Frequency_Hz, and k = 3, where mutual information places AngleOfAttack_deg in the third position instead of ChordLength_m (see Table 2). The raw scores explain the source of this distinction: while NIGSME produces the clearly highest value for Frequency_Hz (0.0923), random forest assigns nearly equal importance to the two variables (0.4283 and 0.3898), and mutual information places ChordLength_m (0.0874) below that of AngleOfAttack_deg (0.1091) (see Table 3).

NIGSME outperforms the other methods at both decision points, and this superiority is consistent across all three partitions. At k = 1, the NIGSME-based model achieves an R^2^ value of 0.1395 on the test set, while the random forest model remains at R^2^ = 0.1123 (Figure 6b); this difference becomes even more pronounced on the validation set (0.1601 vs. 0.0983; Figure 6c). The error metrics support this finding: the RMSE is 6.0746 versus 6.1698 on the test set (Figure 6q), while the MAE is 4.8484 versus 5.0761 (Figure 6h). This difference becomes quantitatively stronger at k = 3; while NIGSME yields R^2^ = 0.4413 on the test set, the mutual information ranking remains at R^2^ = 0.3437 (Figure 6b), and the difference widens to 0.5175 versus 0.3533 on the validation set (Figure 6c). In parallel, on the validation set, the RMSE decreases by 13.6% (5.4897 vs. 4.7420; Figure 6r), the MAE decreases by 16.6% (4.4839 vs. 3.7416; Figure 6i), and the MAPE by 0.59 points (3.0079% vs. 3.6025%; Figure 6l). The adjusted R^2^ values follow the same pattern as R^2^ (Figure 6d–f); since the number of variables (*p* = 5) is small relative to the sample size, the penalty term does not alter the ranking. Furthermore, the fact that the test and validation performance exceed the training performance (R^2^ = 0.5381 and 0.5522, respectively, compared to 0.5028 at k = 5) indicates that there is no overfitting and that the selected subsets are generalizable. As a result, NIGSME does not lag behind any of the comparison methods on this dataset and produces the ranking with the best performance at both k values where a distinction was observed.

The raw importance scores and rankings reported in Table 2 and Table 3 were calculated using all observations in the dataset; these tables reflect the variable importance structure of each method across the entire dataset. In the prediction performance evaluation presented in Figure 6, however, the feature rankings for each method were recalculated using only the training set (70%); neither the test nor the validation sets were used during either the variable selection or model estimation phases. This distinction was intentional: basing the variable selection on the entire dataset would have introduced information leakage into the test and validation metrics and would have artificially inflated the generalization performance. The scores derived from the training set may show minor numerical differences compared to those calculated using the entire sample; however, in the Airfoil Self-Noise dataset, the two calculations produced identical variable rankings.

### 3.2. Data 2—AirQualityUCI Dataset

**Figure 7:**
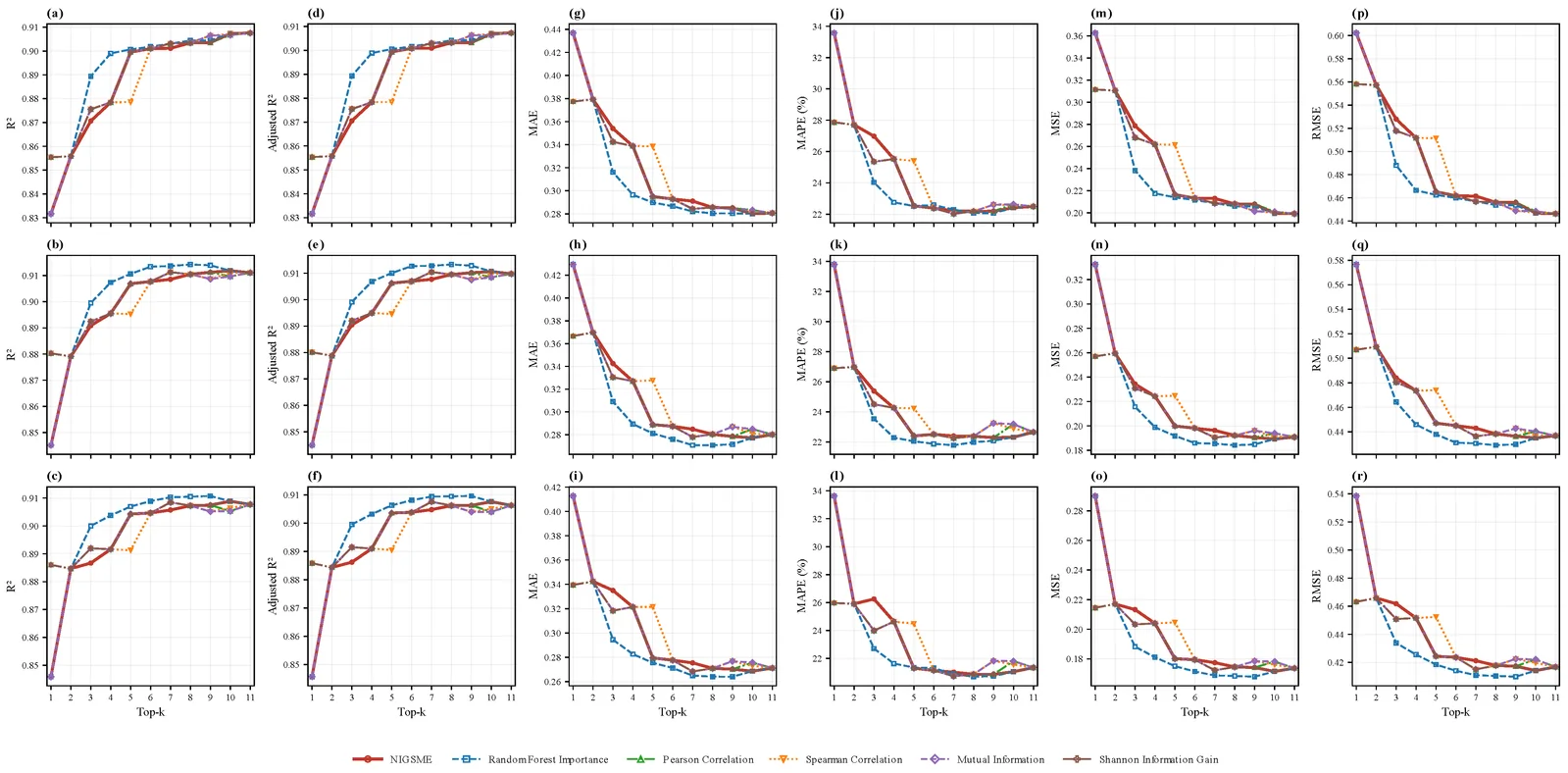
OLS estimation performance of NIGSME and comparison methods on top-k feature subsets in the AirQualityUCI dataset. (Note: Rows correspond to partitions and columns to performance metrics; panel letters progress from top to bottom across each column. Top row (**a**,**d**,**g**,**j**,**m**,**p**)—training partition; middle row (**b**,**e**,**h**,**k**,**n**,**q**)—test partition; bottom row (**c**,**f**,**i**,**l**,**o**,**r**)—validation set. The columns, from left to right, contain the R^2^, adjusted R^2^, MAE, MAPE, MSE, and RMSE metrics, respectively. In each panel, the horizontal axis represents the number of selected features (top-k), and the vertical axis represents the corresponding performance metric. The feature rankings are calculated only on the training set).

Figure 7 presents the performance of OLS models built using top-k subsets generated by six feature selection methods on the AirQualityUCI dataset (*n* = 5000, *p* = 11) on the training (70%, *n* = 3500), test (15%, *n* = 750), and validation (15%, *n* = 750) partitions. In this 11-variable dataset, the decision boundary is much wider compared to Airfoil; however, the methods still produce identical results at certain k values. The overlap of all curves at k = 2 stems from the methods selecting the same pair—PT08.S2(NMHC) and C6H6(GT)—as the first two variables; this overlap in the rankings can be seen directly in Table 4. Similarly, at k = 11, since all methods are reduced to the full model, the performance metrics are, by definition, equal. The first point where a distinction is observed is k = 1: while the Pearson, Spearman, and Shannon Information Gain methods select C6H6(GT) as the top single predictor, NIGSME, random forest, and mutual information select PT08.S2(NMHC) (see Table 4). The raw scores reveal the reason for this divergence: while NIGSME assigns the highest value (0.5781) to PT08.S2(NMHC), the correlation-based metrics favor C6H6(GT) because it exhibits a stronger linear relationship with the target (Pearson r = 0.9292) (see Table 5). This single selection difference creates a performance advantage in favor of the correlation-based cluster at k = 1 (test R^2^ = 0.8803 vs. 0.8452; Figure 7b).

In the intermediate k range (k = 3 to k = 9), the difference between the methods follows a small but consistent pattern. In this range, the random forest importance yields the highest performance; this is because this method adds a strong complementary variable early on by placing NOx(GT) in the third position, whereas NIGSME places PT08.S5(O3) in the third position (see Table 4). For example, at k = 4, random forest achieves an R^2^ value of 0.9074 on the test set, while NIGSME remains at an R^2^ level of 0.8955 (Figure 7b); the difference is also evident in the error metrics (test RMSE of 0.4459 versus 0.4736, Figure 7q; MAPE of 22.28% versus 24.26%, Figure 7k). However, these differences are quite limited in absolute terms: within the range in question, all six methods cluster within the R^2^ = 0.89–0.91 band, and the largest difference between the best method and NIGSME does not exceed 0.012 (Figure 7b,c). The adjusted R^2^ values follow the same pattern as R^2^ (Figure 7d–f), and the fact that all three partitions remain close to one another indicates that there is no overfitting. When these findings are evaluated together, it is observed that NIGSME demonstrates a performance comparable to the built-in correlation- and information-based filters on the AirQualityUCI dataset; it does not fall to the position of the weakest method at any k value; however, specifically for this dataset, random forest importance holds a slight advantage in the medium k range.

### 3.3. Data 3—BodyFat Dataset

**Figure 8:**
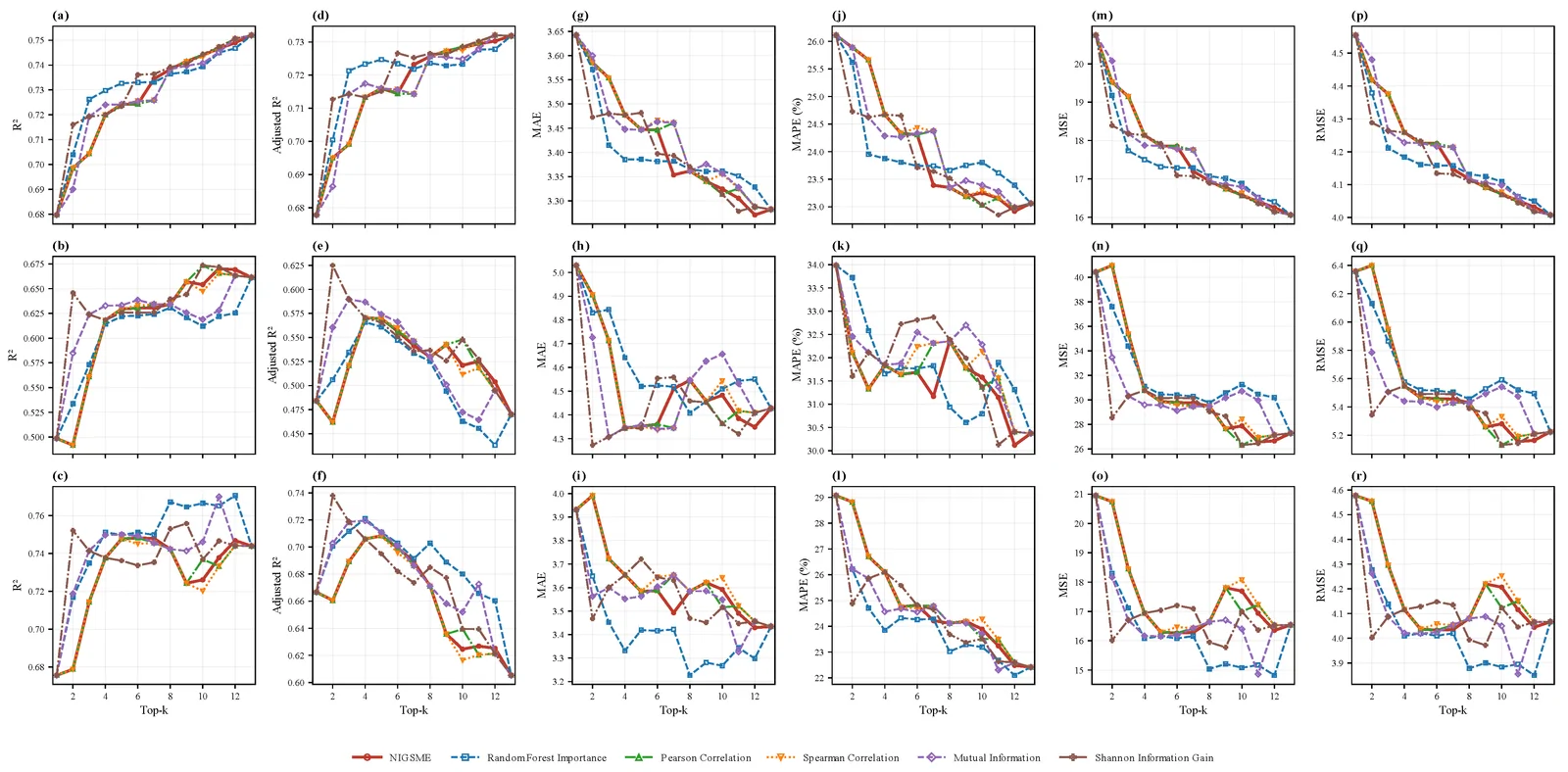
OLS estimation performance of NIGSME and comparison methods on top-k feature subsets in the BodyFat dataset. (Note: Rows correspond to partitions and columns to performance metrics; panel letters progress from top to bottom across each column. Top row (**a**,**d**,**g**,**j**,**m**,**p**)—training partition; middle row (**b**,**e**,**h**,**k**,**n**,**q**)—test partition; bottom row (**c**,**f**,**i**,**l**,**o**,**r**)—validation set. The columns, from left to right, contain the R^2^, adjusted R^2^, MAE, MAPE, MSE, and RMSE metrics, respectively. In each panel, the horizontal axis represents the number of selected features (top-k), and the vertical axis represents the corresponding performance metric. Feature rankings are calculated only on the training set.)

Figure 8 presents the performance of OLS models built using the top-k subsets of the six methods on the BodyFat dataset (*n* = 249, *p* = 13) on the training (69.88%, *n* = 174), test (14.86%, *n* = 37), and validation (15.26%, *n* = 38) partitions. A defining structural feature of this dataset is that the NIGSME ranking almost entirely overlaps with the Pearson and Spearman correlation rankings: all three methods select the variables Abdomen (1), Chest (2), Hip (3), Weight (4), and Thigh (5) within the same top five (see Table 6); consequently, the curves of these methods largely overlap across all panels (Figure 8a–r). Since all methods select Abdomen at k = 1, their performance is identical (test R^2^ = 0.4985; Figure 8b). The most pronounced divergence among the comparison methods stems from random forest importance; while this method ranks the Height variable second, the correlation-based measures place the same variable near the bottom (13th in the Pearson and Spearman rankings; see Table 6 and Table 7). This difference reflects the known sensitivity of tree-based importance measures to correlated variables and leads to the random forest method systematically diverging from the other methods.

For this dataset, the differences between the methods are both small and inconsistent across splits; this inconsistency is a direct result of the single split relying on small test (*n* = 37) and validation (*n* = 38) sets. In the test split, NIGSME yields the highest performance at k = 1 and k = 9 (R^2^ = 0.6569), k = 12, and k = 13 (Figure 8b); in the middle k range, the Shannon Information Gain and mutual information take a slight lead (for example, at k = 2, Shannon R^2^ = 0.6458 versus NIGSME 0.4918). In contrast, the pattern shifts in the validation set, and random forest takes the lead in the medium-to-high k range (for example, at k = 8, R^2^ = 0.7671 versus NIGSME 0.7423; Figure 8c). The fact that the superiority observed in one split is not replicated in the others—particularly that fandom forest’s advantage in the validation set does not appear in the test set—indicates that no method demonstrates consistent superiority on this dataset. The adjusted R^2^ values exhibit the expected decline at high k values due to the small training set (*n* = 174) relative to the 13 variables (Figure 8d–f), suggesting that models built with a small number of variables are sufficient for this dataset. Overall, NIGSME performs consistently and stably, closely matching the performance of established correlation filters; it never ranks as the weakest method for any partition or k value. The sensitivity of the findings to partitioning reveals that the comparisons in this dataset support the general equivalence of the methods rather than strong claims of superiority.

### 3.4. Data 4—Meteorology Dataset

**Figure 9:**
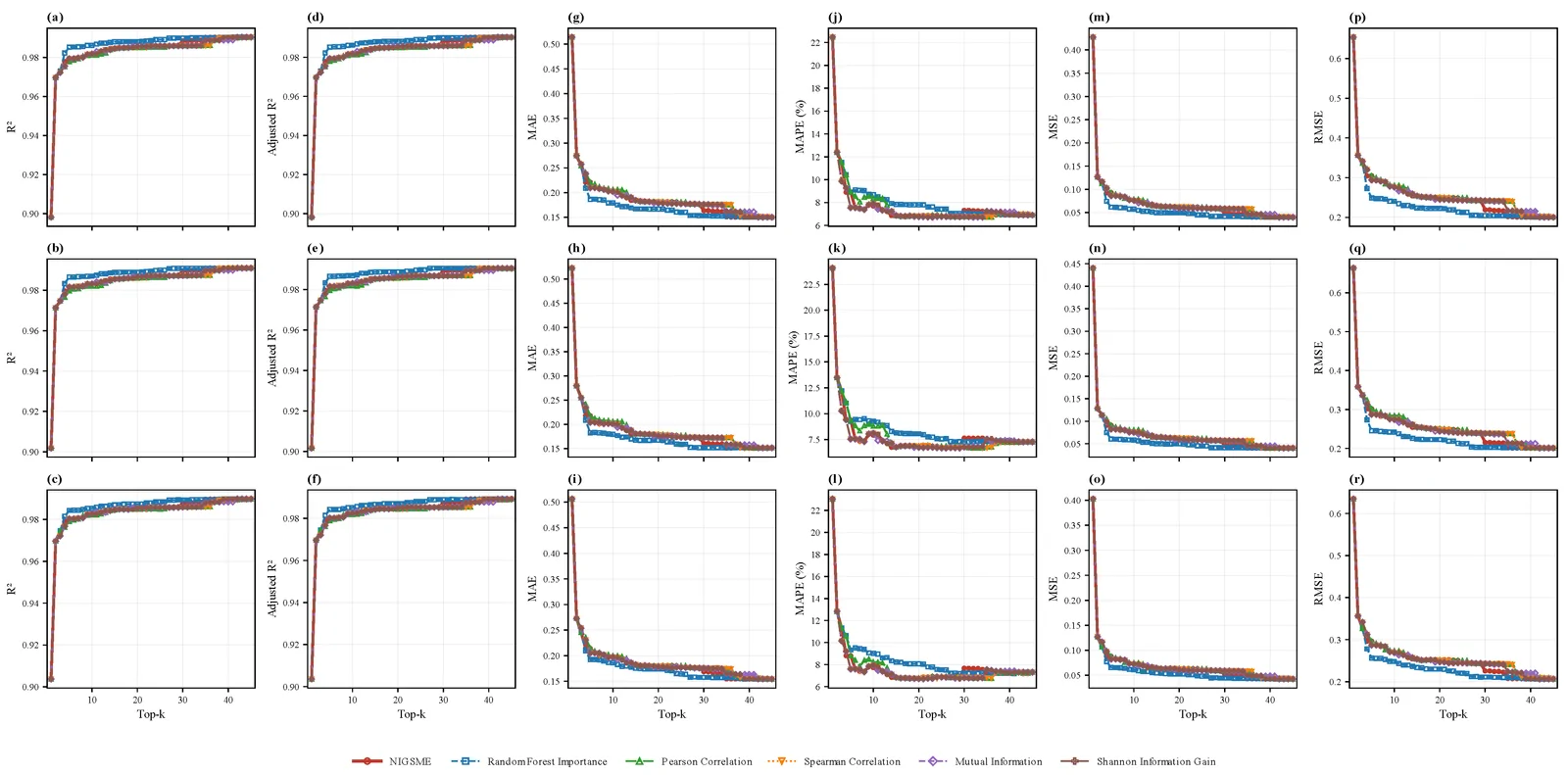
OLS estimation performance of NIGSME and comparison methods on top-k feature subsets in the Meteorology dataset. (Note: Rows correspond to partitions and columns to performance metrics; panel letters progress from top to bottom across each column. Top row (**a**,**d**,**g**,**j**,**m**,**p**)—training partition; middle row (**b**,**e**,**h**,**k**,**n**,**q**)—test partition; bottom row (**c**,**f**,**i**,**l**,**o**,**r**)—validation set. The columns, from left to right, contain the R^2^, adjusted R^2^, MAE, MAPE, MSE, and RMSE metrics, respectively. In each panel, the horizontal axis represents the number of selected features (top-k), and the vertical axis represents the corresponding performance metric. Feature rankings are calculated only on the training set).

Figure 9 presents the performance of ECC models built using the top-k subsets of the six methods on the Meteorology dataset (*n* = 5000, *p* = 45) across the training (70%, *n* = 3500), test (15%, *n* = 750), and validation (15%, *n* = 750) partitions. For this dataset of 45 variables, the performance is determined by two dominant variables: all the methods rank the variables “shortwave_radiation_sum” and “vapour_pressure_deficit_max” in the top two positions (see Table 8); consequently, all curves overlap at k = 1 and k = 2, and the model achieves a test R^2^ of 0.9714 already with just the second variable (Figure 9b). NIGSME assigns the highest scores to these two variables (0.6082 and 0.5446, respectively; see Table 9). At k = 3, the methods remain practically identical (test R^2^ = 0.9747), and the actual divergence begins in the k ≥ 4 range. The most pronounced difference in ranking among the methods is observed for the “sunshine_duration” variable, where the entropy-based measures diverge from linear correlations: while NIGSME ranks this variable sixth, the Pearson correlation ranks it fourteenth (see Table 8). Random forest, on the other hand, produces a completely different ranking—for example, placing relative_humidity_2m_mean in fourth place and various wind variables in the top ranks—and structurally diverges from the other five methods.

In terms of performance, while random forest yields the highest values in the k ≥ 4 range, its superiority is quantitatively negligible: The largest R^2^ difference between random forest and NIGSME in the test set is only 0.0069 (k = 5; Figure 9b), and all six methods are concentrated within a narrow, high range of R^2^ = 0.97–0.99. Within this range, NIGSME is the strongest method among the comparison filters; in the test set, it exceeds the Pearson correlation coefficient for 25 out of 45 k values, and for the remainder, it is equal to or very close to it (Figure 9b). The same pattern is maintained in the validation set (26/45; Figure 9c), indicating that this finding is not due to set-specific variability. Since all methods are reduced to the full model at k = 45, the performance metrics converge to R^2^ ≈ 0.991 on the test set. The adjusted R^2^, MAE, MAPE, and RMSE panels confirm the same pattern (Figure 9d–r); the fact that the test and validation curves are very close to each other indicates that the selected subsets are highly generalizable. In conclusion, in the Meteorology dataset, NIGSME performs at least as well as all correlation- and information-based filters and outperforms them for most k values; the slight numerical advantage of random forest is too limited to be of practical significance.

### 3.5. Data 5—Concrete Dataset

**Figure 10:**
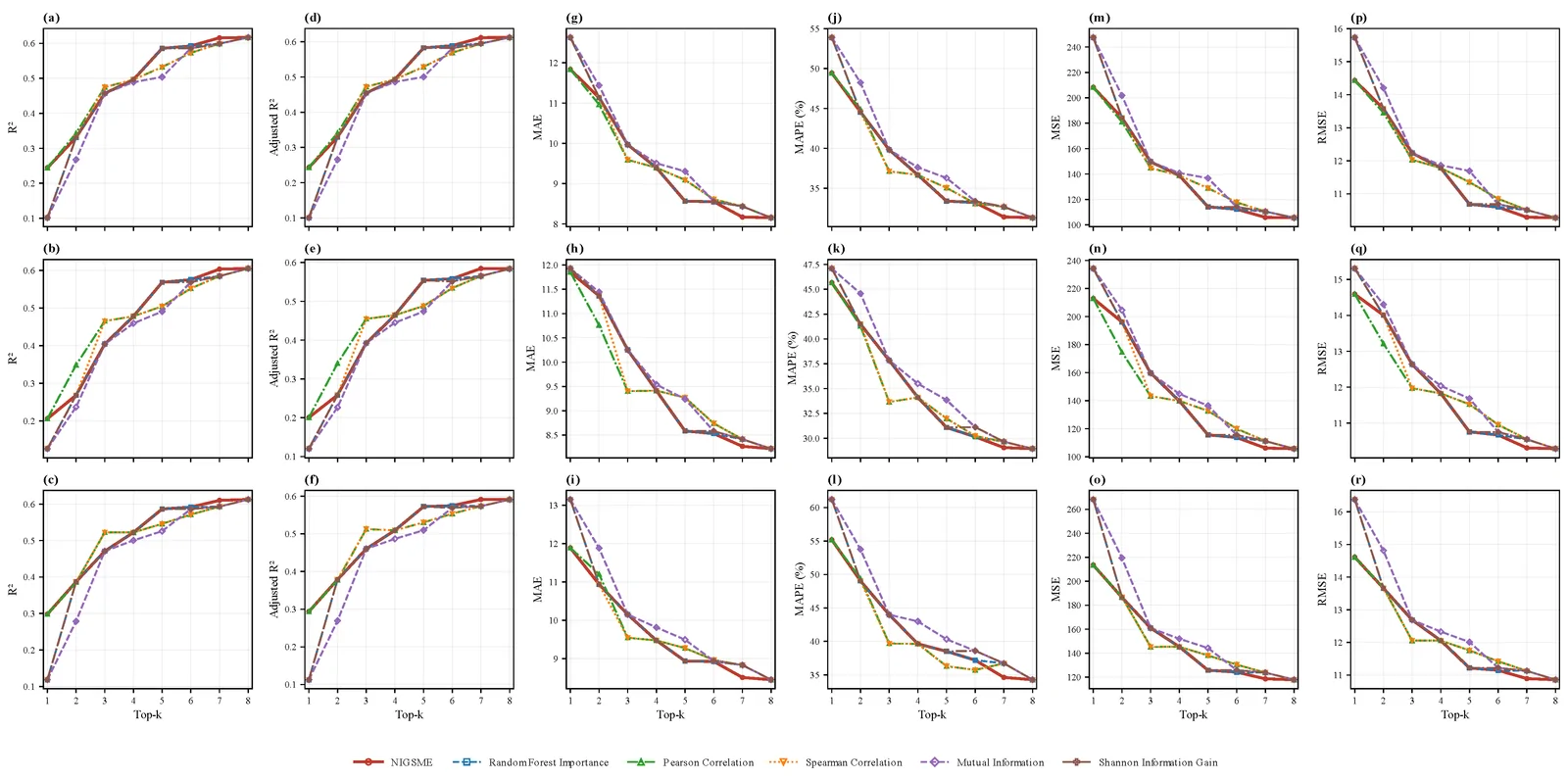
OLS estimation performance of NIGSME and comparison methods on top-k feature subsets in the Concrete dataset. (Note: Rows correspond to partitions and columns to performance metrics; panel letters progress from top to bottom across each column. Top row (**a**,**d**,**g**,**j**,**m**,**p**)—training partition; middle row (**b**,**e**,**h**,**k**,**n**,**q**)—test partition; bottom row (**c**,**f**,**i**,**l**,**o**,**r**)—validation set. The columns, from left to right, contain the R^2^, adjusted R^2^, MAE, MAPE, MSE, and RMSE metrics, respectively. In each panel, the horizontal axis represents the number of selected features (top-k), and the vertical axis represents the corresponding performance metric. Feature rankings are calculated only on the training set.)

Figure 10 presents the performance of the OLS models built using the top-k subsets of the six methods on the Concrete dataset (*n* = 1030, *p* = 8) across the training (70%, *n* = 721), test (14.95%, *n* = 154), and validation (15.05%, *n* = 155) splits. Since concrete’s compressive strength is a nonlinear target, the absolute R^2^ values remain moderate (≈ 0.61 for the full model); however, the relative comparison among methods is not affected by this. The distinction becomes apparent in the very first variable: while NIGSME and the Pearson correlation select “Cement” as the most informative single variable, random forest, Spearman, mutual information, and Shannon Information Gain rank “Age” first (see Table 10). This difference in selection is directly tested using the out-of-sample performance, and NIGSME’s preference is confirmed: at k = 1, the Cement-based model yields R^2^ = 0.2057 on the test set, while the Age-based model remains at only R^2^ = 0.1259 (Figure 10b); a similar and even larger difference is observed on the validation set (0.2982 vs. 0.1184; Figure 10c). The raw scores support this distinction; NIGSME assigns the highest value (0.1112) to “Cement” (see Table 11).

Across the performance curves, NIGSME yields the highest or one of the highest values for most methods. On the test set, NIGSME achieves the best performance at k = 1, 4, 5, 6, 7, and 8; only at k = 2 and k = 3 does the Pearson correlation take the lead by a narrow margin (0.4042 vs. 0.4655 at k = 3; Figure 10b). The validation set largely replicates this pattern, with NIGSME remaining the best or co-best performer for all values of k except k = 3 (Figure 10c)—this consistency between the two sets indicates that the superiority is not due to set-specific fluctuations. The most striking finding emerges at k = 7: At this point, NIGSME achieves a test R^2^ of 0.6036, nearly matching the performance of the full eight-variable model (R^2^ = 0.6054), while all five other methods remain at R^2^ = 0.5851 (Figure 10b). This means that NIGSME can maintain the performance of the full model by excluding a single variable, thereby concretely demonstrating the method’s advantage in model parsimony. The MAE, MAPE, and RMSE panels (Figure 10g–r) confirm the same pattern. As a result, on the Concrete dataset, NIGSME outperforms both the correlation- and information-based filters as well as random forest, demonstrating the best overall performance and most clearly highlighting the distinctive contribution of the proposed method on this dataset.

### 3.6. Data 6—WineQualityWhite Dataset

**Figure 11:**
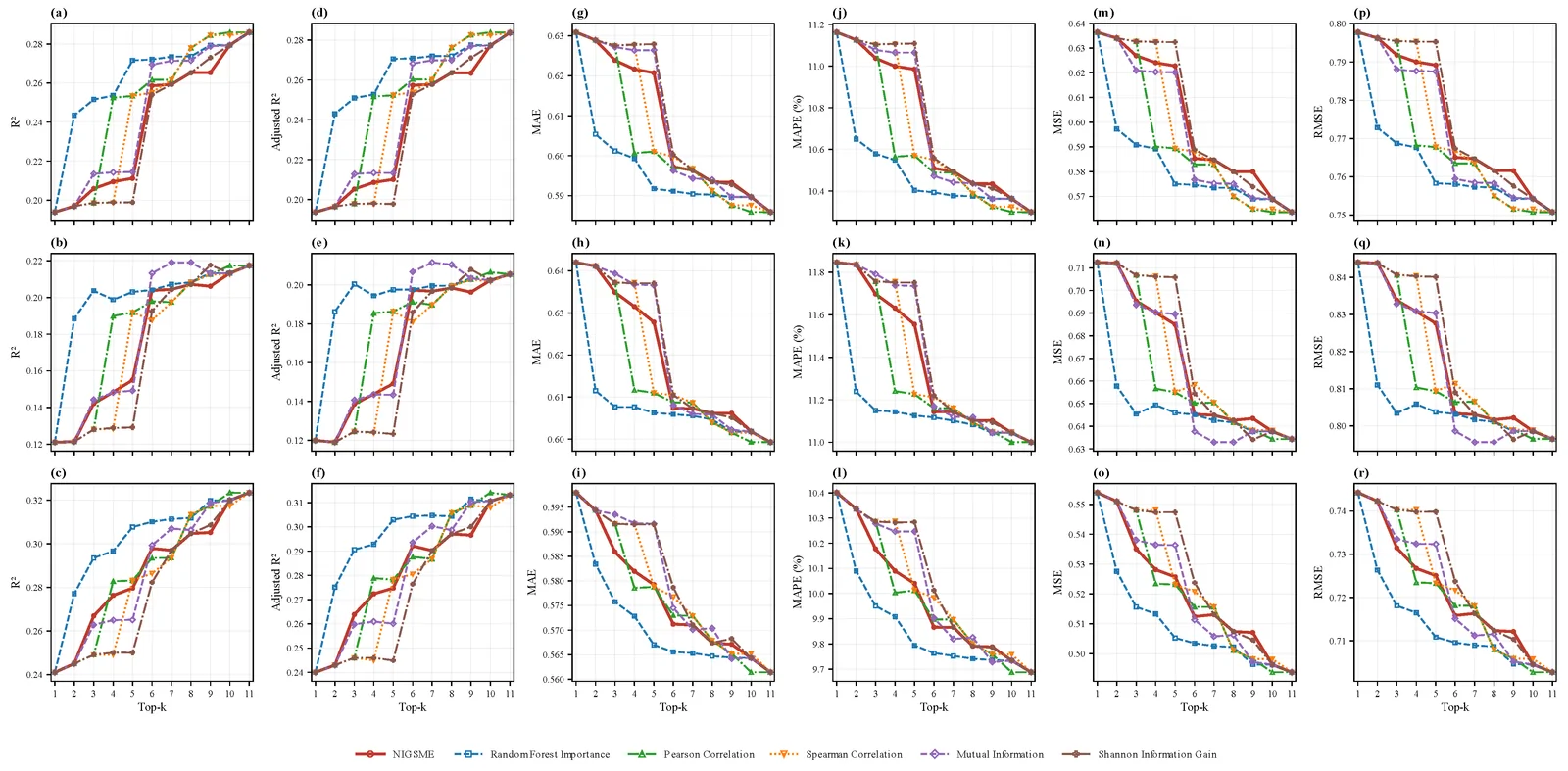
OLS estimation performance of NIGSME and comparison methods on top-k feature subsets in the WineQualityWhite dataset. (Note: Rows correspond to partitions and columns to performance metrics; panel letters progress from top to bottom across each column. Top row (**a**,**d**,**g**,**j**,**m**,**p**)—training partition; middle row (**b**,**e**,**h**,**k**,**n**,**q**)—test partition; bottom row (**c**,**f**,**i**,**l**,**o**,**r**)—validation set. The columns, from left to right, contain the R^2^, adjusted R^2^, MAE, MAPE, MSE, and RMSE metrics, respectively. In each panel, the horizontal axis represents the number of selected features (top-k), and the vertical axis represents the corresponding performance metric. Feature rankings are calculated only on the training set.)

Figure 11 presents the performance of OLS models built using top-k subsets of the six methods on the WineQualityWhite dataset (*n* = 4898, *p* = 11) across the training (70.01%, *n* = 3429), test (%15.01, *n* = 735), and validation (%14.99, *n* = 734) sets. Due to the ordinal and narrowly ranged nature of the target variable (quality score), this dataset can be explained only to a limited extent by linear models; even in the full model, the test R^2^ remains at 0.2174 (Figure 11b). Although this low explanatory power makes the differences between methods appear proportionally large, on an absolute scale they all fall within a narrow range. Since all methods selected “alcohol” as the most informative single variable, the performance at k = 1 is identical (test R^2^ = 0.1211; Figure 11b). Differences begin to emerge starting at k = 2: while NIGSME ranks “density” second (in the same direction as Pearson and Spearman), random forest selects “volatile acidity” (see Table 12). The raw scores explain this preference; NIGSME assigns a value of 0.0636 to density and 0.0374 to volatile acidity (see Table 13). A significant finding specific to this dataset is that the adjusted NIGSME calculation produces a positive information gain for all variables (ranging from 0.0168 to 0.0917; see Table 13), thereby preserving the interpretability of the metric across the entire dataset.

In the performance comparison, random forest importance outperforms all other methods in this dataset across the low-to-medium k range (k = 2 to k = 9); for example, at k = 3, it achieves an R^2^ value of 0.2037 on the test set, while NIGSME remains at an R^2^ level of 0.1422 (Figure 11b), and the same pattern is repeated on the validation set (0.2935 vs. 0.2669; Figure 11c). This superiority reflects the expected advantage of a tree-based importance measure for a target with a nonlinear and interactive structure. However, the difference closes rapidly as k increases; starting from k ≥ 6, all six methods converge for the test set within the R^2^ = 0.20–0.22 range and, by definition, become equal at k = 11 (R^2^ = 0.2174). Among the filtering methods, NIGSME maintains a balanced position: it performs best at k = 1; remains practically indistinguishable from Pearson, Spearman, and Shannon Information Gain at k = 6 and beyond; and never falls to the position of the weakest method at any k value. The RMSE and MAPE panels yield the same results (Figure 11q,k), and the consistency across the three partitions indicates that the findings are not partition specific. In conclusion, WineQualityWhite serves as an example illustrating the limitations of the proposed metric; it demonstrates that tree-based importance metrics can offer advantages over filter-based approaches in nonlinear target structures, while NIGSME maintains a performance comparable to established filter methods in this context as well.

## 4. Discussion

### 4.1. Evolution of Entropy Measures in Information Theory

Entropy is one of the fundamental concepts in information theory, statistics, physics, and machine learning. Introduced by Shannon to measure uncertainty in a probability distribution, entropy quantifies the mean information content of a random variable and forms the foundation of modern information theory. Although the Shannon entropy is still widely used, generalized measures of entropy have been developed because a single formulation is not always sufficient to account for the various types of uncertainty that arise in complex systems. In particular, systems involving long-range interactions, non-equilibrium states, multifractal structures, power law behavior, or non-additive information composition can be more appropriately represented by alternative measures, such as the Rényi, Tsallis, Kaniadakis, Abe, and Sharma–Mittal entropies [7].

One of the first parametric generalizations was proposed by Rényi, who defined the α-order entropy for finite probability distributions. This formulation incorporates the *α* deformation parameter into the model while preserving some fundamental properties of Shannon entropy—such as symmetry and additivity—for independent distributions. The Shannon entropy is obtained in the limit as *α* → 1 [17].

Another important development was the Tsallis entropy, proposed as a generalization of the Boltzmann–Gibbs statistic. This entropy includes the deformation parameter *q*; in the limit as *q* → 1, it reduces to the Boltzmann–Gibbs/Shannon form. It has become one of the fundamental components of non-expansive statistical mechanics. Due to its non-additive structure, it is well-suited to modeling systems involving strong correlations, non-ergodic behavior, and long-range interactions. Tsallis-based approaches have been applied to many complex phenomena, such as turbulence, anomalous diffusion, biological systems, financial markets, and physical systems with long-range interactions [18].

Despite their importance, the Rényi and Tsallis entropies represent different paths of generalization, and neither can be considered a direct extension of the other. The Sharma–Mittal entropy overcomes this limitation by providing a two-parameter framework that encompasses both formulations as limit cases. Specifically, the Sharma–Mittal entropy reduces to the Rényi entropy as *r* → 1, to the Tsallis entropy as *r* → *q*, and to the Shannon entropy at the corresponding classical parameter limits [16,17,18,19].

The importance of the Sharma–Mittal entropy does not stem solely from its unifying nature; it also offers a broader capacity for generalization. Beck [20] considers the Sharma–Mittal entropy to be among the most important generalized measures of information used in characterizing complex systems, and notes that this framework encompasses various well-known entropy measures, such as the Tsallis, Kaniadakis, and Abe entropies, as special cases. Similarly, Ilić et al. [22] classify the Sharma–Mittal entropy within a broad family of generalized entropic forms and emphasize its relationship with other deformed logarithmic and trace-form entropy structures.

Recent studies have also examined the mathematical behavior of generalized entropies under different probability distributions. Bodnarchuk et al. analyzed the Shannon, Rényi, generalized Rényi, Tsallis, Sharma–Mittal, and modified Shannon entropies for various distributions, such as the gamma, chi-square, exponential, Laplace, and log-normal distributions. Their findings showed that entropy values and parameter-dependent behaviors vary depending on both the distribution families and the entropy formulations; therefore, they emphasized the need to consider the probabilistic structure of the data when selecting an entropy measure [40].

In general, entropy theory has evolved from the classical Shannon/Boltzmann–Gibbs formulation into a broad family of generalized information-theoretic tools. Within this development, the Sharma–Mittal entropy stands out as a comprehensive formulation that, thanks to its two-parameter structure, can encompass major measures, such as the Rényi, Tsallis, and Shannon entropies at appropriate limits, and offer flexibility in modeling complex probabilistic systems [16,19,40].

### 4.2. Sharma–Mittal Entropy as a Unifying Two-Parameter Framework

The Sharma–Mittal entropy family provides a comprehensive framework that unifies various generalized entropy measures under a common mathematical structure. Unlike single-parameter entropy formulations, the Sharma–Mittal entropy is determined by two parameters. This structure allows for the control of both the deformation of the probability distribution and the degree of non-expansion within the same framework. Therefore, the Sharma–Mittal entropy can be regarded not only as an alternative entropy measure but also as a general framework that explains the relationships among different entropy families [16].

The fundamental significance of the Sharma–Mittal entropy lies in its ability to encompass well-known entropy measures as special or limiting cases. Under appropriate parameter choices, it can be reduced to the Rényi entropy, the Tsallis entropy, and ultimately the Shannon/Boltzmann–Gibbs entropy. In this respect, it serves as a two-parameter mathematical bridge between entropy measures that were previously treated as separate generalizations. However, its thermostatsistical interpretation has also been critically discussed in the literature [20,21].

Masi showed that the Sharma–Mittal entropy can be interpreted as a natural extension of generalized logarithmic and exponential formalisms and that it provides a consistent framework for explaining the relationships between the Rényi, Tsallis, and Shannon/Boltzmann–Gibbs entropies [19]. In contrast, Aktürk et al. argued that the physical interpretation of this entropy must be handled with caution. The authors showed that a free energy-based interpretation of the Sharma–Mittal relation may not always be valid unless specific thermodynamic assumptions are satisfied [21].

The broader theoretical significance of the Sharma–Mittal entropy has also been highlighted in classifications of generalized entropies. Ilić et al. evaluated the Sharma–Mittal entropy among the two main families of two-parameter entropies in statistical physics and information theory and demonstrated that various entropy formulations can be derived from this family through appropriate parameter choices [22]. Similarly, Bodnarchuk et al. analyzed the Shannon, Rényi, generalized Rényi, Tsallis, Sharma–Mittal, and modified Shannon entropies under different probability models and showed that parameter-dependent entropy behavior changes according to the distribution family [40].

This two-parameter structure provides the Sharma–Mittal entropy with significant flexibility in characterizing uncertainty. While the Shannon entropy assigns a single fixed uncertainty value to a probability distribution, the Sharma–Mittal entropy defines a parametric entropy surface that varies depending on the parameters *α* and *β*. This allows the entropy measure to be sensitive in different ways to distributional properties, such as concentration, tail behavior, low-probability events, and the dominance of high-probability events [19,22,40].

For this reason, the Sharma–Mittal entropy provides a flexible theoretical foundation for modeling uncertainty in complex data structures. Its two-parameter structure provides theoretical support for the use of Sharma–Mittal-based measures of information in data analysis, feature selection, and machine learning applications.

### 4.3. Extensions of Sharma–Mittal Entropy in Information Theory

The importance of the Sharma–Mittal entropy does not stem solely from its role as a generalized measure of uncertainty. Thanks to its two-parameter structure and its ability to encompass various classical entropy measures as limiting cases, it provides a flexible foundation for extending information-theoretic concepts beyond the Shannon framework. From this perspective, the Sharma–Mittal entropy provides a theoretical foundation for generalized forms of concepts such as uncertainty reduction, information transfer, and dependency measurement.

One of the key research areas is the generalization of measures of information transfer. In classical information theory, mutual information expresses the extent to which uncertainty about a random variable is reduced by observing another variable. Since this concept is closely related to information gain, it is also of central importance in feature selection, decision trees, and other learning models. Extending this concept beyond the Shannon entropy allows for the evaluation of feature importance under more flexible uncertainty structures, particularly in cases where the data distribution cannot be adequately represented by a single, fixed measure of entropy.

One of the significant contributions in this area was made by Ilić and Djordjević. The authors defined *α*-*q* mutual information and *α*-*q* channel capacity as Sharma–Mittal-based measures of information transfer [41]. The proposed framework satisfies fundamental information-theoretic properties, such as non-negativity, boundedness by input and output Sharma–Mittal entropies, consistency with perfect transmission, and zero information transfer in completely degrading channels [41]. Furthermore, with appropriate parameter choices, the measure can be reduced to the classical mutual information, Rényi mutual information, or Tsallis mutual information. These properties indicate that the Sharma–Mittal entropy is not only a descriptive uncertainty measure but also a potential basis for generalized dependence and information-transfer metrics.

Another important extension relates to generalized distance/disjointness measures, which are used to quantify differences between probability distributions and are widely used in information theory, statistics, and information geometry. Nielsen and Nock derived closed-form expressions for Rényi and Tsallis entropies and divergence measures for distributions belonging to exponential families and showed that these measures can be expressed in terms of the log-normalizing function of the relevant family [42]. Although this study focused on the Rényi and Tsallis families, it is theoretically important because both are special cases or limiting cases of the Sharma–Mittal framework.

This divergence-based perspective extends entropy-based thinking from a measure of uncertainty to a distributional comparison. In a Sharma–Mittal-based framework, the two-parameter form can enable the evaluation of differences between probability distributions under varying sensitivity conditions. The Sharma–Mittal entropy has also been extended to generalized entropy measures, and its two-parameter framework has been used to investigate concavity properties in diffusion processes [43].

Overall, these developments indicate that the Sharma–Mittal entropy has evolved into a more comprehensive information-theoretic framework that extends many concepts related to the Shannon entropy. The existence of generalized mutual information, divergence measures, and entropy powers provides a theoretical foundation for research into Sharma–Mittal-based learning algorithms and information-driven feature evaluation approaches.

### 4.4. Applications of Sharma–Mittal Entropy Across Scientific Domains

The theoretical flexibility of the Sharma–Mittal entropy has enabled its use in various scientific fields. Although it was initially developed in the context of generalized information theory and statistical physics, it has increasingly been applied in areas where uncertainty, distributional complexity, and nonclassical information structures are significant.

In machine learning, Koltcov et al. proposed a framework based on Sharma–Mittal entropy to evaluate topic modeling performance [44]. Their work addressed two fundamental problems in probabilistic topic modeling: determining the optimal number of topics and selecting the appropriate hyperparameters. The findings demonstrated that the Sharma–Mittal entropy can simultaneously reflect both model quality and semantic stability, and that it offers a more comprehensive evaluation metric compared to traditional perplexity-based assessments.

Another application can be found in the field of engineering design. Ahmed et al. used the Sharma–Mittal entropy to measure design diversity and assess the extent to which the design space was explored during the concept generation process [45]. The authors proposed a family of Sharma–Mittal-based design diversity metrics and introduced the Herfindahl–Hirschman Index for Design (HHID) metric. The experimental results demonstrated that the HHID is more consistent with human evaluations and exhibits higher sensitivity to changes in design variety.

The Sharma–Mittal entropy has also been used in decision tree and ensemble learning methods. Ignatenko et al. investigated information gain criteria based on Rényi, Tsallis, and Sharma–Mittal entropies in random forest algorithms for classification and regression tasks [46]. By replacing the classical Shannon-based split criterion with generalized entropy-based information gain formulations, they improved the prediction performance on six benchmark datasets. Among the entropy measures tested, the Sharma–Mittal information gain frequently yielded the best results.

This result is particularly important in terms of feature selection, as information gain is closely related to the assessment of variable importance in tree-based learning. Although Ignatenko et al. focused on random forest architecture rather than a direct, independent feature selection method, their findings provide direct evidence that Sharma–Mittal-based information gain can serve as a practical alternative to Shannon-based metrics in machine learning systems [46].

Applications can also be found in reliability theory and lifetime analysis. Mohamed and Sakr investigated the cumulative residual Sharma–Taneja–Mittal entropy and developed nonparametric estimation procedures for it [47]. Their work analyzed the properties related to stochastic comparisons, hazard rate functions, mean residual life functions, equilibrium random variables, and record values [47]. Similarly, Sfetcu et al. proposed an alternative measure of cumulative residual Sharma–Taneja–Mittal entropy and examined its mathematical properties [48]. These studies indicate that Sharma–Mittal-derived entropy measures are applicable to stochastic modeling and reliability analysis, and not only to information theory.

Beyond statistics and machine learning, the Sharma–Mittal entropy has also been studied in the fields of thermodynamics and cosmology. Abreu and Ananias Neto analyzed the generalized second law of thermodynamics for various non-Gaussian entropy formalisms, including the Sharma–Mittal entropy, in the context of visible horizon thermodynamics [49]. Their findings suggest that parameter constraints may be necessary to preserve thermodynamic consistency in extended entropy formulations [49]. Similarly, Naeem and Bibi developed a corrected Friedmann equation based on the Sharma–Mittal entropy, contributing to the interpretation of generalized entropy structures in cosmological models [50].

In general, the literature shows that the Sharma–Mittal entropy has evolved from a theoretical generalized entropy formulation into a multidisciplinary analytical framework. Its application areas include topic modeling and random forest learning in machine learning; the measurement of design diversity in engineering; residual life and reliability analysis; and thermodynamic and cosmological modeling. However, despite this growing body of literature, applications to feature selection remain relatively limited; there is a notable research gap, particularly regarding studies involving continuous probability distributions and information measures based on density estimation [44,46,49].

### 4.5. Entropy-Based Feature Selection and Information Gain

Feature selection is one of the fundamental preprocessing steps in machine learning, pattern recognition, data mining, and statistical learning. Its primary purpose is to identify the relevant variables while removing irrelevant, redundant, or noisy variables. This reduces dimensionality, decreases computational complexity, improves prediction performance, and enhances a model’s interpretability [1,2,3,4].

The increasing prevalence of high-dimensional datasets has heightened the importance of feature selection methods. In fields such as gene expression analysis, text classification, image processing, network security, and sensor- or web-based data, there may be a large number of variables, and a significant portion of these variables may be irrelevant or redundant. Including such variables in a model can reduce prediction accuracy, increase computational cost, and lead to overfitting [1,2,3,4].

Feature selection methods are generally classified as filter, wrapper, and embedded approaches. Filter methods evaluate features using statistical or data-driven criteria, independent of a specific learning algorithm. Wrapper methods evaluate subsets of features based on model performance, while embedded methods perform feature selection during model training. Among these approaches, filter methods are particularly important because they are computationally efficient, model independent, and suitable for pre-selecting features in high-dimensional datasets [1,2,3,4].

Criteria based on information theory constitute an important group of suitability measures within filter-based feature selection. Measures based on information gain, mutual information, entropy reduction, and dissimilarity evaluate how much information a candidate feature provides about the target variable. Unlike criteria based solely on correlation, these methods rely on uncertainty and statistical dependence structures; therefore, they can also capture informative relationships that cannot be adequately represented by linear correlation [9,10,11,12].

Traditionally, information gain is derived from the Shannon entropy and measures the reduction in uncertainty regarding the target variable after a predictor variable has been observed. This approach has been widely used in decision tree-based learning, and feature splits have been selected based on entropy-based information gain criteria. However, the Shannon entropy represents only a specific formulation of uncertainty. For this reason, generalized entropy families, such as those proposed by Rényi, Tsallis, and Sharma–Mittal, have recently been investigated as alternative foundations for information gain in machine learning [46].

Ignatenko et al. proposed information gain formulations based on the Rényi, Tsallis, and Sharma–Mittal entropies for random forest structures. Their findings showed that generalized entropy-based information gain metrics can improve prediction performance compared to classical Shannon-based approaches in both classification and regression tasks. In particular, the Sharma–Mittal information gain demonstrated promising performance across multiple benchmark datasets and showed that parametric entropy families can provide more flexible partitioning criteria for complex data structures [46].

These findings were significant in that they demonstrated that the Sharma–Mittal information gain is practically applicable in machine learning algorithms. In previous applications, Sharma–Mittal entropy was mostly used as a descriptive, evaluative, or theoretical measure in fields such as subject modeling, design diversity, reliability analysis, thermodynamics, and cosmology. In contrast, its use in the context of information gain represents a more direct integration of this entropy family into prediction-oriented learning systems [46].

However, the existing Sharma–Mittal information gain approaches are largely limited to decision tree construction and random forest splitting procedures. Furthermore, entropy values depend on the selected Sharma–Mittal parameter combinations. Since different parameter settings can highlight different aspects of uncertainty, the resulting information gain scores may also vary depending on these choices. This parameter sensitivity remains a significant methodological challenge for generalized entropy-based feature evaluation [21,22,46].

Therefore, although the Sharma–Mittal information gain has shown promising predictive potential, there is still a need for methods that reduce the dependence on fixed parameter choices and extend entropy-based feature evaluation to information measures based on continuous probability distributions and density estimation.

### 4.6. Continuous Entropy Estimation and Density-Based Approaches

When variables are continuous, estimating quantities based on entropy and information theory becomes more difficult. In discrete structures, entropy can be calculated from observed probability masses or their empirical estimates. In contrast, continuous entropy measures depend on probability density functions; therefore, estimating entropy-based quantities requires either an estimate of the density or a direct estimate of the integral functionals associated with the density.

For this reason, entropy estimation has become an important research topic in statistics, information theory, and machine learning. The primary goal is to develop reliable estimators that can directly approximate entropy-related functionals from observed data, particularly for continuous variables.

Källberg et al. examined statistical inference procedures for Rényi entropy functionals and related entropy-like measures for both discrete and continuous distributions. In their work, estimators based on overlapping or ε-close observations in independent samples were used, and asymptotic properties such as consistency and asymptotic normality were established. This study was significant in that it demonstrated that entropy-type quantities can be treated as integral functionals of probability densities and can be estimated directly from continuous observations [51].

Källberg and Seleznjev further developed this approach by examining entropy-type integral functionals of densities and proposed U-statistic estimators based on ε-near vector observations. They obtained results regarding consistency and asymptotic normality under conditions of weak integrability and smoothness. These findings demonstrated that quantities related to generalized entropy can be estimated for continuous probability distributions without discretizing the data [52].

Alternative approaches have also been proposed to avoid explicit density estimation. Sánchez-Giraldo et al. defined entropy measures from data using infinitely divisible kernels and positive-definite matrices. This framework defines entropy-like functionals on normalized Gram matrices and extends this approach to kernel-based conditional entropy and mutual information. In this respect, it is important for learning problems involving continuous data representations [53].

In general, these studies indicate a shift from discretization-based approaches in entropy calculations toward continuous and data-driven entropy estimation frameworks. These approaches are particularly important for datasets with naturally continuous variables, as they avoid arbitrary binning and better preserve the data’s inherent distributional structure. Therefore, density functional and kernel-based entropy estimation methods provide a useful theoretical foundation for developing entropy-based feature selection criteria for continuous variables.

### 4.7. Limitations of Discretization-Based Entropy Methods

Although entropy-based feature selection methods are widely used, many approaches based on classical information theory are naturally defined for discrete probability distributions. Therefore, when analyzing continuous variables, these variables are often converted into discrete categories before calculating the entropy, mutual information, or information gain.

Discretization divides the range of values of a continuous variable into a finite number of intervals and represents the observations using these interval labels. While this process facilitates probability estimation, it introduces significant limitations. First, discretization may alter the original distributional structure of the variable. Fine-grained information in continuous observations can be lost when the number of intervals is small, whereas too many intervals may lead to sparse probability estimates and unstable entropy values.

Second, entropy estimates may become sensitive to the chosen binning strategy. Different choices regarding the number of bins, bin boundaries, or binning algorithms can yield different information gain values on the same dataset. This creates an additional source of variability that is not directly related to the intrinsic information content of the variables.

Third, discretization often requires subjective design decisions. Researchers must choose a partitioning scheme before entropy calculations, and these choices may affect feature rankings and reduce the reproducibility of entropy-based analyses.

For these reasons, continuous entropy estimation methods have sparked increased interest in the development of non-discretization information theory frameworks. Density functional estimators based on ε-near observations and U-statistics allow for the direct estimation of entropy-related quantities from continuous distributions [51,52]. Kernel-based approaches, on the other hand, offer another alternative that yields entropy-like measures directly from data representations without requiring explicit probability density estimates [53]. Taken together, these developments support the development of entropy-based learning criteria that can operate on continuous variables without relying on discretization procedures [54,55,56].

When the findings from the six datasets are evaluated together, it is observed that the proposed volumetric metric is largely consistent with classical filters, but this consistency does not remain at the same level across all dependency structures. The points where the divergence between methods intensifies emerge in the variables where the strength of linear relationships diverges from the entropic dependence; a typical example of this is the ranking of the `sunshine_duration` variable in the Meteorology dataset, which was placed sixth by NIGSME but fourteenth by the Pearson correlation. In terms of prediction performance, NIGSME outperformed all comparison methods in the Concrete dataset and achieved full model performance with seven variables, providing the advantage of model simplification; it was the strongest among the filter approaches in the Meteorology dataset; and in the Airfoil Self-Noise dataset, it produced the best subset at both points where the rankings diverged. In contrast, in the WineQualityWhite dataset, random forests clearly outperformed other methods in the low-to-medium k range of variable importance; this reflects the expected advantage of tree-based metrics over filter-based approaches given the ordinal and interactive nature of the target. The overall pattern suggests that the proposed metric complements rather than replaces established methods; in particular, it provides discriminative information in continuous data structures where nonlinear but non-monotonic dependencies exist. The ranking equivalence of the three indices further indicates that they should be positioned not as competing selectors but as different reporting scales of a single metric.

## 5. Conclusions

In this study, a feature-ranking framework that does not rely on discretization for continuous target and explanatory variables and is independent of parameter selection was proposed. The conditional and gain surfaces of the Sharma–Mittal entropy were defined in the *α*-*β* plane; the volumes lying beneath these surfaces were calculated using two-dimensional numerical integration to obtain the PICSME, PIGSME, and NIGSME indices. Densities were generated using a Gaussian kernel estimation; the problem of negative information gain that can arise in continuous variables was resolved by maintaining a common bandwidth in the target direction in both the marginal and joint estimations and by renormalizing the conditional densities. Thus, positive and interpretable scores were generated for all variables across all six datasets.

The theoretical properties of the framework were formally analyzed. It was shown that the proposed gain yields exactly zero in the case of independence, reduces to classical mutual information at the Shannon corner, and is non-negative at this limit; it was also demonstrated that, in the general parameter region, the sign depends on the balance between weak dependence and the Jensen gap arising from the convexity of the transformation. Furthermore, since the marginal entropy volume of the target is independent of the candidate variable in a fixed dataset, it was proven that PIGSME and NIGSME are exact decreasing affine transformations of PICSME; it was shown that the fact that the three indices produce the same ranking is not an empirical finding but a mathematical necessity. Therefore, the three metrics should be interpreted not as independent selectors, but as absolute, differential, and proportional reporting scales of a single ranking metric.

The experimental evaluation was not limited to the ranking consistency; feature rankings were calculated solely from the training partition, and the out-of-sample prediction performance was measured using least-squares models on top-k subsets. The results show that the proposed metric outperformed all comparison methods on the Concrete dataset and all filter approaches on the Meteorology dataset; it produced the best subset at both points where the rankings diverged on the Airfoil Self-Noise dataset; and it demonstrated performance on par with established methods on the AirQualityUCI and BodyFat datasets. The superiority of random forest variable importance in the low-to-medium k range on the WineQualityWhite dataset reflects the advantage of tree-based metrics in nonlinear and interactive target structures. When evaluated as a whole, the proposed metric never ranked as the weakest method in any dataset; it demonstrated consistent and competitive performance across the different dependency structures.

These findings demonstrate that volumetric entropy measures defined across an entire parameter space provide a feature-ranking tool for continuous variables that is free from uncertainties arising from discretization and parameter selection. The method is positioned not so much as an alternative to existing filters, but rather as a tool that provides complementary information in situations where the linear relationship strength and entropic dependence diverge.

## 6. Limitations

**Figure 12:**
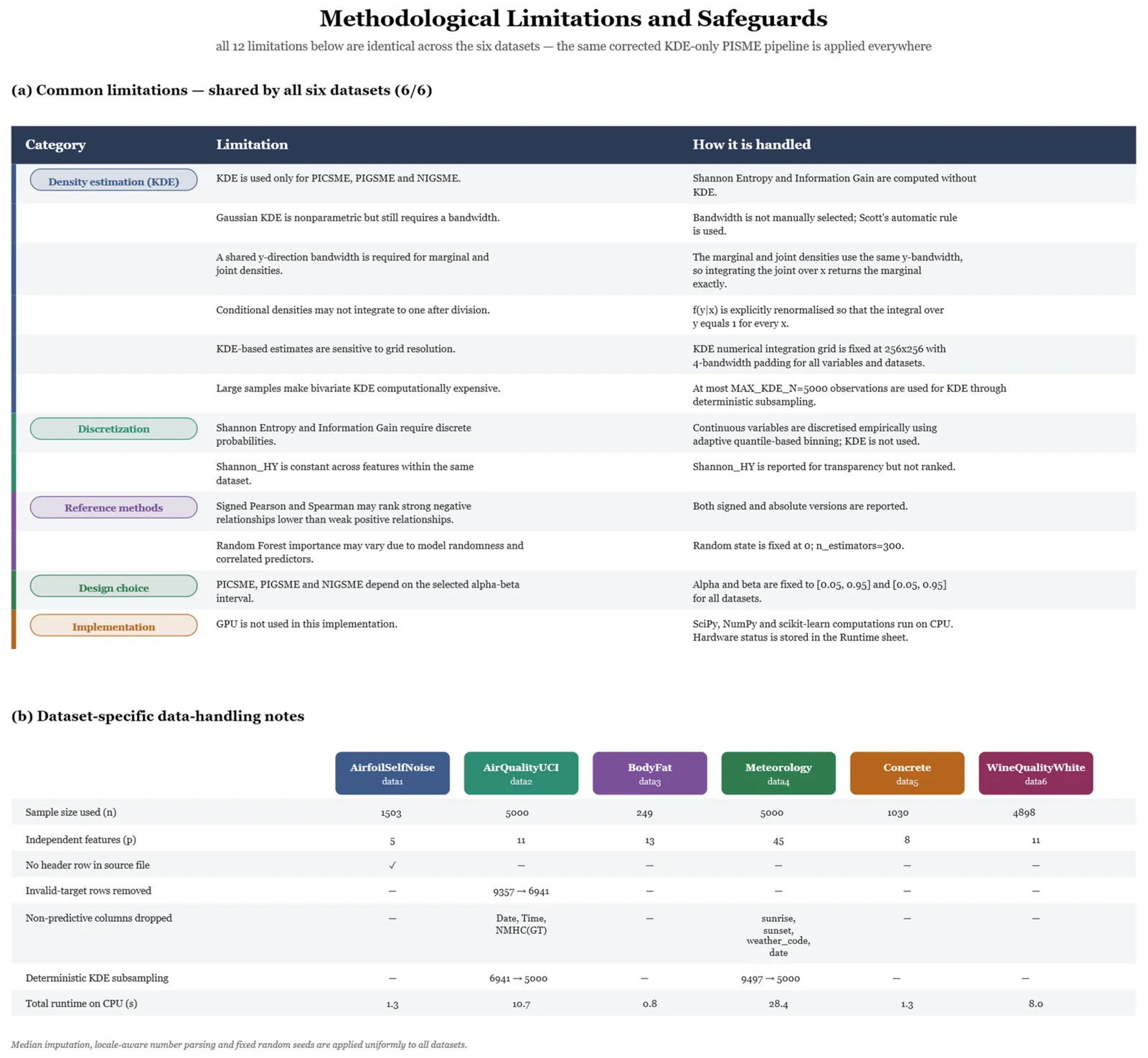
Methodological limitations, assurance measures, and data processing design of the study. Note: (**a**) Methodological limitations common to all six datasets and the standardized assurance measures implemented to mitigate these limitations. (**b**) Information specific to each dataset regarding sample size, number of independent variables, data cleaning, removal of invalid target observations, exclusion of non-predictive variables, deterministic subsampling, and computation time.

The findings of this study should be interpreted within the methodological limitations listed below. These limitations and the measures taken to address them are summarized visually in Figure 12; the data processing steps specific to each dataset, along with the total computation times and the results of the sensitivity analysis regarding design choices, are presented in detail in Appendix D.

First, the density estimation was performed using a Gaussian kernel and the Scott bandwidth rule. Although kernel density estimation is a nonparametric approach, it requires a bandwidth selection, and the resulting entropy estimates may be sensitive to this choice. The use of an automatic rule instead of manually adjusting the bandwidth, and the application of the same protocol across all datasets, aimed to eliminate this sensitivity as a source of methodological variation (Figure 12; Appendix D). The impact of this design decision was also tested via a sensitivity analysis; it was confirmed that the rankings exhibited high stability across five datasets (Spearman *ρ* ≥ 0.99) under alternative bandwidth selectors (scaled variants of the Silverman and Scott rule), while only the smallest dataset, Concrete, exhibited moderate sensitivity to the bandwidth scale (*ρ* = 0.83–0.98); even in this case, the composition of the top-three variables was preserved across all variations (see Appendix D, Table A3). Mass transport beyond the boundary of the Gaussian kernel in variables with limited support may still constitute a source of bias in the estimates.

Second, the results depend on the selected parameter region. The *α* and *β* parameters were constrained to the range [0.05, 0.95] to avoid singularities at the points *α* = 1 and *β* = 1. Since this region excludes the Shannon corner, the criterion operates in a strong deformation regime throughout the entire region. The behavior of volume values under different integration boundaries and different grid resolutions were also examined in the same sensitivity analysis, and it was shown that the rankings remained completely stable with respect to these choices (*ρ* = 1.00 in all datasets) (Appendix D).

Third, due to computational cost, deterministic subsampling was applied to large datasets during the two-variable density estimation stage. The effect of subsampling selection on the rankings was tested using multiple seed values, and it was confirmed that the rankings were robust to seed selection (Appendix D). The subsampling steps applied for each dataset and the total computation times are summarized in Figure 12 and reported in detail in Appendix D.

Fourth, since the three proposed indices were monotonic transformations of one another, they produced the same rankings. While this is an advantage in terms of consistency, additional work is required—such as cross-dataset standardization, modeling uncertainty in the target entropy, or extension with different objective functions—to enable the three metrics to provide distinct decision information.

Fifth, the evaluation of prediction performance was conducted using a single random split (70% training, 15% test, 15% validation) and least-squares regression. While calculating rankings based solely on the training set prevented information leakage, using a single split could lead to the results being sensitive to the split, particularly in datasets with small sample sizes; the differences in patterns observed between the test and validation sets in the BodyFat dataset point to this. Furthermore, the use of a linear model limited the assessment of nonlinear dependencies.

Finally, the comparison methods were limited to five common metrics; wrapper and embedded selection approaches were excluded. Computations were performed on the central processing unit (CPU), and graphics processing unit (GPU) acceleration was not used; the runtime per dataset and total CPU times for each method are presented in Figure 12 and Appendix D.

## Figures and Tables

**Table 1:** Analytical profile of the datasets and the data processing steps applied.

Dataset	Data Size (N)	Feature Size	Target Variable	Application Area	Data Processing Summary
Airfoil Self-Noise	1503	5	SoundPressureLevel_dB	Acoustics/aerodynamics	Five continuous aerodynamic variables and the target variable SoundPressureLevel_dB were used directly in the analysis.
AirQualityUCI	7674	11	CO(GT)	Weather quality	Observations with an invalid target variable were removed; the date, time, and NMHC(GT) columns were excluded from the predictor set. During the density estimation phase, 5000 observations were used with deterministic subsampling.
BodyFat	249	13	BodyFat	Body composition	The analysis file, which consists of body composition measurements, included 13 continuous explanatory variables.
Meteorology	9497	45	et0_fao_evapotranspiration	Hydrometeorology	The variables “date,” “sunrise,” “sunset,” and “weather_code” were excluded from the model. For the density estimate, a deterministic subsampling of 5000 observations was applied.
Concrete	1030	8	Concrete compressive strength (MPa)	Materials engineering	Eight mixture/age variables were used as explanatory variables for the compressive strength target.
WineQualityWhite	4898	11	Quality	Food/wine quality	Physicochemical parameters specific to white wines were used; all observations were directly incorporated into the density-based calculation.

Note: From the initial 9357 observations in the AirQualityUCI dataset, records with invalid target values were removed, leaving 7674 observations. In both the AirQualityUCI and meteorological datasets, a subsampling of 5000 observations was applied only during the KDE phase to limit the cost of density estimation.

**Table 2:** Rankings of derived features for the eight methods on the Airfoil Self-Noise dataset.

Variables	Pearson Correlation	Spearman Correlation	Shannon Information Gain	Mutual Information	Random Forest Importance	NIGSME
Frequency_Hz	1	1	1	1	2	1
SuctionSideDisplacementThickness_m	2	2	3	2	1	2
ChordLength_m	3	3	2	4	3	3
AngleOfAttack_deg	4	4	4	3	4	4
FreeStreamVelocity_m_s	5	5	5	5	5	5

Note: Rank 1 corresponds to the highest importance. NIGSME is listed in descending order. The rank equivalence of the proposed scores under the same target variable and parameter region was also evaluated.

**Table 3:** Raw importance scores of all methods on the Airfoil Self-Noise dataset.

Variables	Pearson Correlation	Spearman Correlation	Shannon Information Gain	Mutual Information	Random Forest Importance	PICSME	PIGSME	NIGSME
Frequency_Hz	−0.3907	−0.3408	0.1810	0.1855	0.3898	2.3042	0.2343	0.0923
AngleOfAttack_deg	−0.1561	−0.1408	0.1182	0.1091	0.0436	2.4274	0.1111	0.0437
ChordLength_m	−0.2362	−0.2430	0.1331	0.0874	0.0954	2.4128	0.1257	0.0495
FreeStreamVelocity_m_s	0.1251	0.1162	0.0696	0.0342	0.0430	2.4713	0.0671	0.0265
SuctionSideDisplacementThickness_m	−0.3127	−0.2798	0.1194	0.1664	0.4283	2.3671	0.1713	0.0675

Note: The raw coefficients marked in the Pearson and Spearman columns are reported. In the rank analysis, the absolute values of both correlation coefficients are used. For PICSME, a lower score indicates a greater reduction in the conditional uncertainty; for PIGSME and NIGSME, a higher score indicates a greater relative gain in information. The scales are interpreted within the dataset.

**Table 4:** Rankings of derived features for the eight methods on the AirQualityUCI dataset.

Variables	Pearson Correlation	Spearman Correlation	Shannon Information Gain	Mutual Information	Random Forest Importance	NIGSME
PT08.S2(NMHC)	2	2	2	1	1	1
C6H6(GT)	1	1	1	2	2	2
PT08.S1(CO)	3	3	3	3	4	3
PT08.S5(O3)	4	4	4	4	7	4
NOx(GT)	5	6	5	5	3	5
PT08.S3(NOx)	6	5	6	6	8	6
PT08.S4(NO2)	8	8	8	8	11	7
NO2(GT)	7	7	7	7	5	8
RH	9	11	10	10	9	9
T	11	10	9	9	6	10
AH	10	9	11	11	10	11

Note: Rank 1 corresponds to the highest importance. NIGSME is listed in descending order. The rank equivalence of the proposed scores under the same target variable and parameter region was also evaluated.

**Table 5:** Raw importance scores of all methods on the AirQualityUCI dataset.

Variables	Pearson Correlation	Spearman Correlation	Shannon Information Gain	Mutual Information	Random Forest Importance	PICSME	PIGSME	NIGSME
PT08.S1(CO)	0.8756	0.8767	0.6938	0.7713	0.0236	1.3163	1.2657	0.4902
C6H6(GT)	0.9292	0.9274	0.9039	1.0480	0.4218	1.1022	1.4798	0.5731
PT08.S2(NMHC)	0.9142	0.9274	0.9039	1.0496	0.4426	1.0893	1.4927	0.5781
NOx(GT)	0.7874	0.7831	0.5658	0.6627	0.0361	1.6032	0.9788	0.3791
PT08.S3(NOx)	−0.7020	−0.8142	0.5454	0.5862	0.0095	1.6185	0.9635	0.3732
NO2(GT)	0.6769	0.7303	0.4260	0.4700	0.0190	1.9007	0.6813	0.2639
PT08.S4(NO2)	0.6329	0.6010	0.2882	0.3593	0.0066	1.8895	0.6926	0.2682
PT08.S5(O3)	0.8552	0.8606	0.6649	0.7157	0.0114	1.3174	1.2646	0.4898
T	0.0157	0.0657	0.0282	0.0395	0.0153	2.4541	0.1279	0.0495
RH	0.0674	0.0209	0.0276	0.0333	0.0074	2.4511	0.1309	0.0507
AH	0.0617	0.0732	0.0169	0.0319	0.0066	2.4913	0.0907	0.0351

Note: The raw coefficients marked in the Pearson and Spearman rows are reported. In the rank analysis, the absolute values of both correlation coefficients are used. For PICSME, a lower score indicates a greater reduction in conditional uncertainty; for PIGSME and NIGSME, a higher score indicates a greater relative gain in information. The scales are interpreted within the dataset.

**Table 6:** Rankings of derived features for the eight methods on the BodyFat dataset.

Variables	Pearson Correlation	Spearman Correlation	Shannon Information Gain	Mutual Information	Random Forest Importance	NIGSME
Abdomen	1	1	1	1	1	1
Chest	2	2	2	2	8	2
Hip	3	4	4	3	10	3
Weight	4	3	3	5	5	4
Thigh	5	5	6	4	11	5
Knee	6	8	7	6	7	6
Neck	8	7	8	8	4	7
Biceps	7	6	5	7	13	8
Forearm	9	9	10	11	12	9
Height	13	13	13	13	2	10
Wrist	10	10	11	10	3	11
Age	11	12	9	9	6	12
Ankle	12	11	12	12	9	13

Note: Rank 1 corresponds to the highest importance. NIGSME is listed in descending order. The rank equivalence of the proposed scores under the same target variable and parameter region was also evaluated.

**Table 7:** Raw importance scores of all methods on the BodyFat dataset.

Variables	Pearson Correlation	Spearman Correlation	Shannon Information Gain	Mutual Information	Random Forest Importance	PICSME	PIGSME	NIGSME
Age	0.2881	0.2689	0.2583	0.0916	0.0243	2.5083	0.1564	0.0587
Weight	0.5987	0.6041	0.4349	0.2700	0.0245	2.2735	0.3912	0.1468
Height	−0.1089	−0.0272	0.1878	0.0000	0.0441	2.4915	0.1732	0.0650
Neck	0.4752	0.4798	0.2599	0.1028	0.0260	2.3881	0.2767	0.1038
Chest	0.6938	0.6674	0.4534	0.3306	0.0219	2.0869	0.5778	0.2168
Abdomen	0.8077	0.8125	0.6126	0.5750	0.7205	1.8464	0.8183	0.3071
Hip	0.6136	0.6037	0.4110	0.2950	0.0206	2.1950	0.4698	0.1763
Thigh	0.5431	0.5348	0.3160	0.2797	0.0180	2.3512	0.3135	0.1177
Knee	0.4928	0.4791	0.3013	0.1958	0.0220	2.3816	0.2831	0.1062
Ankle	0.2516	0.2886	0.2241	0.0367	0.0213	2.5417	0.1230	0.0462
Biceps	0.4756	0.4825	0.3450	0.1868	0.0147	2.4048	0.2599	0.0975
Forearm	0.3418	0.3797	0.2523	0.0674	0.0155	2.4704	0.1943	0.0729
Wrist	0.3278	0.3000	0.2289	0.0732	0.0267	2.5016	0.1631	0.0612

Note: The raw coefficients marked in the Pearson and Spearman rows are reported. In the rank analysis, the absolute values of both correlation coefficients are used. For PICSME, a lower score indicates a greater reduction in conditional uncertainty; for PIGSME and NIGSME, a higher score indicates a greater relative gain in information. The scales are interpreted within the dataset.

**Table 8:** Rankings of derived features for the eight methods on the Meteorology dataset.

Variables	Pearson Correlation	Spearman Correlation	Shannon Information Gain	Mutual Information	Random Forest Importance	NIGSME
shortwave_radiation_sum	1	1	1	1	1	1
vapour_pressure_deficit_max	2	2	2	2	2	2
temperature_2m_max	3	4	4	4	3	3
temperature_2m_mean	5	5	5	5	6	4
soil_temperature_0_to_7cm_mean	4	6	6	6	8	5
sunshine_duration	14	3	3	3	15	6
soil_temperature_7_to_28cm_mean	6	7	8	8	12	7
daylight_duration	7	8	7	7	9	8
relative_humidity_2m_mean	8	9	9	9	4	9
temperature_2m_min	10	11	11	12	14	10
soil_temperature_0_to_100cm_mean	9	10	10	11	18	11
relative_humidity_2m_min	13	12	12	10	24	12
wet_bulb_temperature_2m_max	12	13	13	14	21	13
wet_bulb_temperature_2m_mean	15	15	15	15	30	14
soil_temperature_28_to_100cm_mean	11	14	14	13	17	15
wet_bulb_temperature_2m_min	16	16	16	16	29	16
relative_humidity_2m_max	17	17	17	17	11	17
cloud_cover_mean	19	19	19	18	20	18
dew_point_2m_max	18	18	18	19	27	19
pressure_msl_max	21	22	21	23	34	20
pressure_msl_mean	23	25	24	25	37	21
dew_point_2m_mean	22	21	22	22	33	22
pressure_msl_min	24	26	25	24	31	23
cloud_cover_max	20	20	23	26	26	24
soil_moisture_0_to_7cm_mean	27	23	20	20	22	25
cloud_cover_min	25	24	26	27	40	26
precipitation_hours	28	29	28	30	41	27
soil_moisture_7_to_28cm_mean	29	28	27	21	25	28
dew_point_2m_min	26	27	29	32	28	29
wind_gusts_10m_mean	43	45	36	35	7	30
surface_pressure_min	45	44	33	34	36	31
precipitation_sum	30	30	31	31	43	32
surface_pressure_mean	41	39	35	33	39	33
rain_sum	31	33	34	36	42	34
surface_pressure_max	35	36	37	37	38	35
wind_speed_10m_mean	42	38	42	43	5	36
wind_gusts_10m_max	44	43	38	40	13	37
soil_moisture_0_to_100cm_mean	32	34	30	28	32	38
wind_speed_10m_max	38	37	45	45	10	39
soil_moisture_28_to_100cm_mean	36	35	32	29	35	40
snowfall_sum	33	31	39	38	44	41
snowfall_water_equivalent_sum	34	32	40	39	45	42
wind_gusts_10m_min	37	41	43	41	19	43
wind_direction_10m_dominant	39	40	41	42	23	44
wind_speed_10m_min	40	42	44	44	16	45

Note: Rank 1 corresponds to the highest importance. NIGSME is listed in descending order. The rank equivalence of the proposed scores under the same target variable and parameter region was also evaluated.

**Table 9:** Raw importance scores of all methods on the Meteorology dataset.

Variables	Pearson Correlation	Spearman Correlation	Shannon Information Gain	Mutual Information	Random Forest Importance	PICSME	PIGSME	NIGSME
temperature_2m_max	0.9110	0.9186	0.8322	0.9345	0.0223	0.9987	1.1181	0.5282
temperature_2m_min	0.8511	0.8489	0.6192	0.6574	0.0005	1.2719	0.8450	0.3992
precipitation_sum	−0.3084	−0.3781	0.1393	0.1632	0.0000	1.9632	0.1536	0.0726
rain_sum	−0.2792	−0.3412	0.1197	0.1227	0.0000	1.9852	0.1316	0.0622
snowfall_sum	−0.1803	−0.3449	0.0671	0.0831	0.0000	2.0400	0.0768	0.0363
precipitation_hours	−0.4047	−0.3947	0.1654	0.1764	0.0000	1.8938	0.2230	0.1053
sunshine_duration	0.8054	0.9215	0.8662	0.9867	0.0004	1.1316	0.9852	0.4654
daylight_duration	0.8753	0.8804	0.7226	0.7911	0.0013	1.2079	0.9089	0.4294
wind_speed_10m_max	0.1014	0.1232	0.0400	0.0245	0.0010	2.0314	0.0854	0.0404
wind_gusts_10m_max	0.0101	0.0532	0.0765	0.0798	0.0006	2.0078	0.1090	0.0515
wind_direction_10m_dominant	−0.0864	−0.0872	0.0643	0.0629	0.0002	2.0454	0.0714	0.0338
shortwave_radiation_sum	0.9485	0.9619	1.1176	1.2836	0.8417	0.8294	1.2874	0.6082
temperature_2m_mean	0.8995	0.9003	0.7733	0.8374	0.0051	1.0657	1.0511	0.4966
cloud_cover_mean	−0.6578	−0.6663	0.3512	0.3752	0.0002	1.6768	0.4400	0.2079
cloud_cover_max	−0.5771	−0.6110	0.2627	0.2599	0.0001	1.8085	0.3083	0.1457
cloud_cover_min	−0.4656	−0.5345	0.2386	0.2553	0.0000	1.8635	0.2533	0.1197
dew_point_2m_mean	0.5703	0.6059	0.2642	0.3025	0.0001	1.8068	0.3100	0.1465
dew_point_2m_max	0.6732	0.7101	0.3619	0.3721	0.0001	1.6778	0.4390	0.2074
dew_point_2m_min	0.4287	0.4524	0.1581	0.1510	0.0001	1.9440	0.1729	0.0817
relative_humidity_2m_mean	−0.8585	−0.8646	0.6678	0.7376	0.0075	1.2414	0.8755	0.4136
relative_humidity_2m_max	−0.7251	−0.7164	0.3736	0.4013	0.0007	1.6327	0.4841	0.2287
relative_humidity_2m_min	−0.8058	−0.8463	0.6155	0.6815	0.0001	1.3137	0.8032	0.3794
pressure_msl_mean	−0.5197	−0.5147	0.2506	0.2645	0.0001	1.7997	0.3171	0.1498
pressure_msl_max	−0.5758	−0.5762	0.2762	0.2918	0.0001	1.7751	0.3417	0.1614
pressure_msl_min	−0.4841	−0.4802	0.2392	0.2702	0.0001	1.8083	0.3085	0.1457
surface_pressure_mean	−0.0629	−0.0875	0.1172	0.1429	0.0001	1.9705	0.1463	0.0691
surface_pressure_max	−0.1443	−0.1543	0.1069	0.1099	0.0001	1.9862	0.1306	0.0617
surface_pressure_min	−0.0094	−0.0463	0.1230	0.1263	0.0001	1.9613	0.1555	0.0735
wind_speed_10m_mean	0.0604	0.0928	0.0598	0.0546	0.0060	1.9926	0.1242	0.0587
wind_speed_10m_min	−0.0678	−0.0798	0.0429	0.0360	0.0003	2.0549	0.0619	0.0293
wind_gusts_10m_mean	−0.0224	0.0442	0.1145	0.1253	0.0017	1.9538	0.1630	0.0770
wind_gusts_10m_min	−0.1030	−0.0840	0.0542	0.0703	0.0003	2.0422	0.0747	0.0353
vapour_pressure_deficit_max	0.9150	0.9399	0.9469	1.0862	0.1055	0.9640	1.1528	0.5446
wet_bulb_temperature_2m_mean	0.8047	0.8258	0.5520	0.5748	0.0001	1.3850	0.7318	0.3457
wet_bulb_temperature_2m_max	0.8145	0.8414	0.5777	0.6087	0.0002	1.3500	0.7668	0.3622
wet_bulb_temperature_2m_min	0.7810	0.7964	0.4899	0.5394	0.0001	1.4663	0.6505	0.3073
soil_temperature_0_to_7cm_mean	0.9014	0.8974	0.7568	0.8340	0.0013	1.1021	1.0147	0.4793
soil_temperature_7_to_28cm_mean	0.8877	0.8852	0.7057	0.7594	0.0007	1.1788	0.9380	0.4431
soil_temperature_28_to_100cm_mean	0.8318	0.8360	0.5707	0.6196	0.0003	1.3869	0.7299	0.3448
soil_temperature_0_to_100cm_mean	0.8549	0.8561	0.6240	0.6704	0.0003	1.3118	0.8050	0.3803
soil_moisture_0_to_7cm_mean	−0.4275	−0.5380	0.3249	0.3622	0.0002	1.8282	0.2886	0.1364
soil_moisture_7_to_28cm_mean	−0.3219	−0.4296	0.2274	0.3352	0.0001	1.9034	0.2134	0.1008
soil_moisture_28_to_100cm_mean	−0.1106	−0.1617	0.1362	0.2449	0.0001	2.0329	0.0839	0.0396
soil_moisture_0_to_100cm_mean	−0.1921	−0.2800	0.1483	0.2463	0.0001	2.0178	0.0990	0.0468
snowfall_water_equivalent_sum	−0.1803	−0.3449	0.0671	0.0809	0.0000	2.0400	0.0768	0.0363

Note: The raw coefficients marked in the Pearson and Spearman rows are reported. In the rank analysis, the absolute values of both correlation coefficients are used. For PICSME, a lower score indicates a greater reduction in conditional uncertainty; for PIGSME and NIGSME, a higher score indicates a greater relative gain in information. The scales are interpreted within the dataset.

**Table 10:** Rankings of derived features for the eight methods on the Concrete dataset.

Variables	Pearson Correlation	Spearman Correlation	Shannon Information Gain	Mutual Information	Random Forest Importance	NIGSME
Cement	1	2	3	3	2	1
Age	3	1	1	1	1	2
Water	4	4	4	2	3	3
Superplasticizer	2	3	5	5	5	4
Fine Aggregate	5	6	8	6	6	5
Blast Furnace Slag	7	7	6	7	4	6
Coarse Aggregate	6	5	7	4	7	7
Fly Ash	8	8	2	8	8	8

Note: Rank 1 corresponds to the highest importance. NIGSME is listed in descending order. The rank equivalence of the proposed scores under the same target variable and parameter region was also evaluated.

**Table 11:** Raw importance scores of all methods on the Concrete dataset.

Variables	Pearson Correlation	Spearman Correlation	Shannon Information Gain	Mutual Information	Random Forest Importance	PICSME	PIGSME	NIGSME
Cement	0.4978	0.4776	0.1853	0.3058	0.3222	2.2016	0.2754	0.1112
Blast Furnace Slag	0.1348	0.1625	0.1261	0.1820	0.0809	2.3957	0.0814	0.0329
Fly Ash	−0.1058	−0.0780	0.2574	0.1276	0.0177	2.3973	0.0797	0.0322
Water	−0.2896	−0.3084	0.1672	0.3537	0.1060	2.2550	0.2221	0.0897
Superplasticizer	0.3661	0.3476	0.1610	0.2224	0.0733	2.3277	0.1493	0.0603
Coarse Aggregate	−0.1649	−0.1835	0.1142	0.2558	0.0283	2.3962	0.0808	0.0326
Fine Aggregate	−0.1672	−0.1800	0.1001	0.2118	0.0370	2.3939	0.0831	0.0336
Age	0.3289	0.5960	0.3127	0.3609	0.3346	2.2303	0.2468	0.0996

Note: The raw coefficients marked in the Pearson and Spearman rows are reported. In the rank analysis, the absolute values of both correlation coefficients are used. For PICSME, a lower score indicates a greater reduction in conditional uncertainty; for PIGSME and NIGSME, a higher score indicates a greater relative gain in information. The scales are interpreted within the dataset.

**Table 12:** Rankings of derived features for the eight methods on the WineQualityWhite dataset.

Variables	Pearson Correlation	Spearman Correlation	Shannon Information Gain	Mutual Information	Random Forest Importance	NIGSME
alcohol	1	1	1	2	1	1
density	2	2	2	1	8	2
chlorides	3	3	5	4	7	3
free sulfur dioxide	11	10	8	6	3	4
total sulfur dioxide	5	4	4	3	6	5
volatile acidity	4	5	7	8	2	6
citric acid	10	11	3	7	11	7
fixed acidity	6	7	6	11	10	8
pH	7	6	11	9	4	9
residual sugar	8	8	9	5	5	10
sulphates	9	9	10	10	9	11

Note: Rank 1 corresponds to the highest importance. NIGSME is listed in descending order. The rank equivalence of the proposed scores under the same target variable and parameter region was also evaluated.

**Table 13:** Raw importance scores of all methods on the WineQualityWhite dataset.

Variables	Pearson Correlation	Spearman Correlation	Shannon Information Gain	Mutual Information	Random Forest Importance	PICSME	PIGSME	NIGSME
fixed acidity	−0.1137	−0.0845	0.0671	0.0263	0.0610	2.1009	0.0610	0.0282
volatile acidity	−0.1947	−0.1966	0.0493	0.0470	0.1245	2.0811	0.0808	0.0374
citric acid	−0.0092	0.0183	0.0715	0.0518	0.0596	2.0824	0.0795	0.0368
residual sugar	−0.0976	−0.0821	0.0331	0.0730	0.0699	2.1087	0.0532	0.0246
chlorides	−0.2099	−0.3145	0.0681	0.0825	0.0634	2.0500	0.1119	0.0518
free sulfur dioxide	0.0082	0.0237	0.0419	0.0668	0.1155	2.0581	0.1038	0.0480
total sulfur dioxide	−0.1747	−0.1967	0.0686	0.1014	0.0685	2.0664	0.0955	0.0442
density	−0.3071	−0.3484	0.1225	0.1753	0.0629	2.0245	0.1374	0.0636
pH	0.0994	0.1094	0.0317	0.0466	0.0712	2.1075	0.0544	0.0251
sulphates	0.0537	0.0333	0.0326	0.0386	0.0619	2.1255	0.0364	0.0168
alcohol	0.4356	0.4404	0.1710	0.1473	0.2417	1.9637	0.1982	0.0917

Note: The raw coefficients marked in the Pearson and Spearman rows are reported. In the rank analysis, the absolute values of both correlation coefficients are used. For PICSME, a lower score indicates a greater reduction in conditional uncertainty; for PIGSME and NIGSME, a higher score indicates a greater relative gain in information. The scales are interpreted within the dataset.

**Figure A1:**
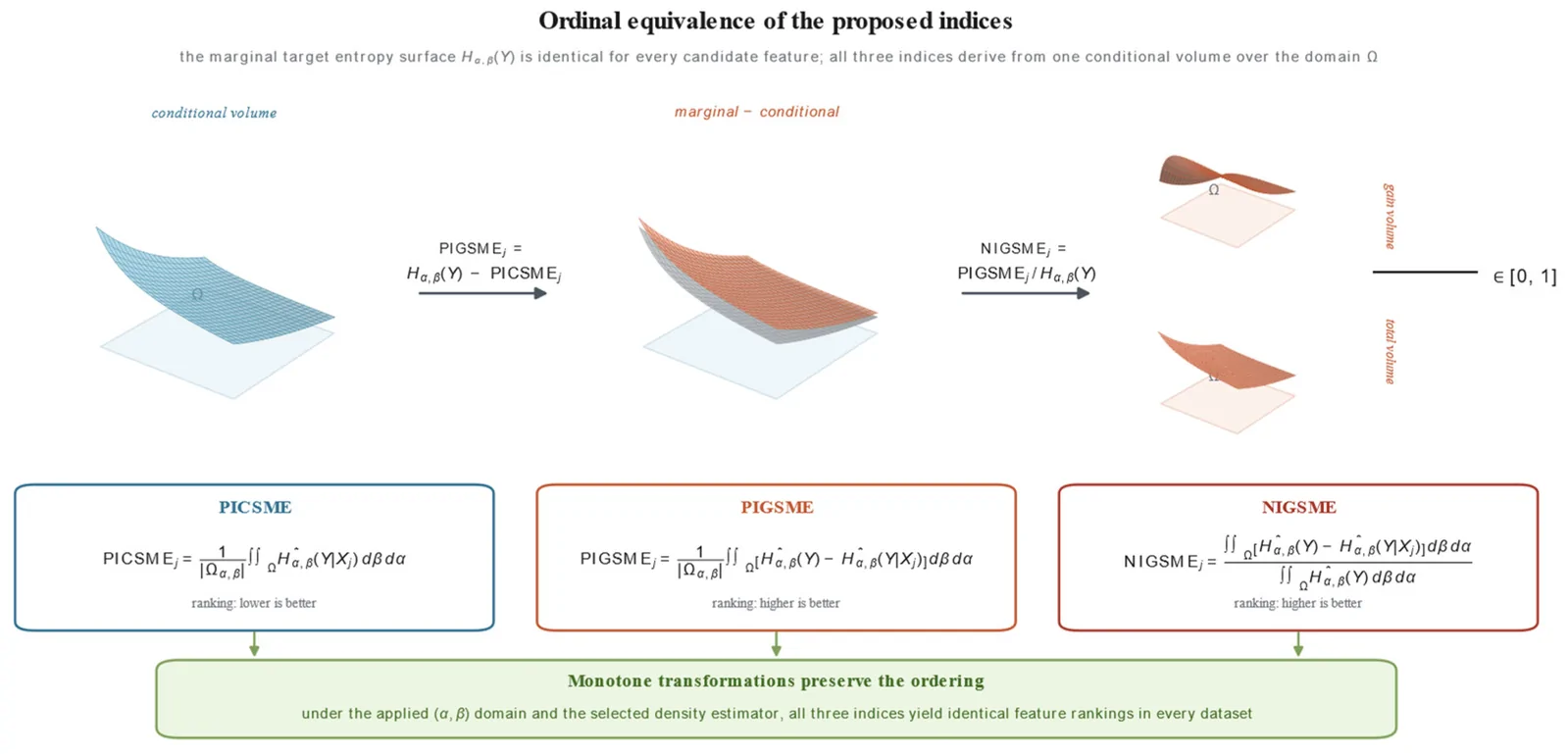
Rank-equivalence relationship among PICSME, PIGSME, and NIGSME. Note: In each dataset, the marginal entropy surface of the target variable is the same for all candidate variables. Therefore, PIGSME is a version of PICSME, from which the constant target volume has been removed; NIGSME is a version of this information gain volume scaled by a constant target volume. Under the applied parameter range and the density estimation method used, these transformations yield the same variable ranking across all datasets. While this observation demonstrates that the three scores do not produce conflicting selections, it also suggests that, for the three metrics to provide distinct decision information in future studies, they need to be extended through cross-dataset standardization, target entropy uncertainty analysis, or the use of different objective functions.

## Data Availability

No new primary data were created in this study. The datasets analyzed in this study are publicly available from their original repositories. The Airfoil Self-Noise, Air Quality, Concrete Compressive Strength, and Wine Quality datasets are available from the UCI Machine Learning Repository. The BodyFat dataset is publicly available from the StatLib/Journal of Statistics Education body fat dataset repository. The meteorological dataset was compiled from publicly available historical weather records obtained through the Open-Meteo Historical Weather API. The preprocessing steps applied to the datasets are described in Section 2. The processed data files, source code, and generated feature-ranking results are available from the corresponding author upon reasonable request.

## Abbreviations

The following abbreviations are used in this manuscript:

DE-PISME-FS	Density-Estimated Parameter-Integrated Sharma–Mittal Entropy Feature Selection
IG	Information Gain
KDE	Kernel Density Estimation
MI	Mutual Information
NIGSME	Normalized Parameter-Integrated Sharma–Mittal Entropy Information Gain
PICSME	Parameter-Integrated Conditional Sharma–Mittal Entropy
PIGSME	Parameter-Integrated Sharma–Mittal Entropy Information Gain
RF	Random Forest
SME	Sharma–Mittal Entropy

## Appendix B

**Table A1:** Notation and assumptions.

Symbol	Meaning
Y	Continuous target variable, density $f_{Y}$
$X_{j}$	j-th candidate explanatory variable, j = 1, …, *p*
$f_{Y,X_{j}}$	Joint density
$f_{Y|X_{j}}$	Conditional density
*α*, *β*	Sharma–Mittal parameters
$D=\left[\alpha_{L},\alpha_{U}\right]\times \left[\beta_{L},\beta_{u}\right]$	Integration region, ${\left[0.05,0.95\right]}^{2}$
|D|	Area of the region, $\left(\alpha_{U}-\alpha_{L}\right)\left(\beta_{U}-\beta_{L}\right)$
$h_{Y},h_{X}$	KDE bandwidths
K(·)	Gaussian kernel

Note: Assumption B1 (Regularity): $f_{Y}$ and $f_{Y,X_{j}}$ are bounded, square integrable, and twice continuously differentiable. Furthermore, for every *α* ∈ $\left[\alpha_{L},\alpha_{U}\right]$, $\int f_{Y}^{\alpha }dy<\infty$ and $\int f_{Y|X_{j}}^{\alpha }dy<\infty$ hold. Assumption B2 (Parameter domain): $D\subset {\left(0,1\right)}^{2}$, with α ≠ 1 and β ≠ 1. This restriction is imposed to prevent the denominators 1 − *α* and 1 − *β* in the Sharma–Mittal definition from vanishing; the interval [0.05, 0.95] provides a safe distance from these singularities.

## Appendix C

**Table A2:** Computational Algorithm.

Step	Description
Step 1	The target Y is standardized; $h_{Y}$ is determined via Equation (12) and ${\hat{f}}_{Y}$ is estimated via Equation (8). The grid over $\left[\min y-4h_{Y},\max y+4h_{Y}\right]$ is divided into $N_{Y}=256$ points.
Step 2	Each candidate $X_{j}$ is standardized; ${\hat{f}}_{Y,X_{j}}$ is estimated via Equation (9), using the same $h_{Y}$ in the *y*-direction (Proposition 2).
Step 3	${\hat{f}}_{X_{j}}$ is obtained via (10); the conditional density is constructed via Equation (11) and renormalized via Equation (14) (Proposition 3).
Step 4	For each α, the integrals $\int {\hat{f}}_{Y}^{\;\alpha }$ and, at every point *x*, $\int {\tilde{f}}_{Y|X_{j}}^{\;\alpha }$ are computed via Equation (15).
Step 5	The surfaces ${\hat{H}}_{\alpha ,\beta }\left(Y\right)$ and ${\hat{H}}_{\alpha ,\beta }\left(Y|X_{j}\right)$ are constructed over a 60×60 (α,β) grid using Equations (1) and (7).
Step 6	The volumes are computed via numerical integration using Equations (18), (20), and (22); applying Equations (19), (21), and (24) yields the three indices.

Note: The overall computational complexity is on the order of $O\left(n\left(N_{y}+N_{x}\right)+N_{y}N_{x}+N_{\alpha }N_{y}N_{x}\right)$ per variables; for datasets with *n* < 5000, *n* is capped via deterministic subsampling.

## Appendix D

**Table A3:** Sensitivity of the NIGSME ranking to the design choices of the estimation procedure.

Variation	Airfoil Self-Noise		AirQualityUCI		BodyFat		Meteorology		Concrete		WineQualityWhite	
	*ρ*	*τ*	*ρ*	*τ*	*ρ*	*τ*	*ρ*	*τ*	*ρ*	*τ*	*ρ*	*τ*
Base (Scott, 60 × 60, [0.05, 0.95])	1.000	1.000	1.000	1.000	1.000	1.000	1.000	1.000	1.000	1.000	1.000	1.000
Bandwidth: Silverman	1.000	1.000	1.000	1.000	1.000	1.000	1.000	0.996	0.976	0.929	1.000	1.000
Bandwidth: Scott ×0.7	1.000	1.000	0.991	0.964	0.995	0.974	0.997	0.970	0.929	0.857	0.991	0.964
Bandwidth: Scott ×1.3	1.000	1.000	1.000	1.000	0.995	0.974	0.999	0.986	0.833	0.714	1.000	1.000
Grid: 30 × 30	1.000	1.000	1.000	1.000	1.000	1.000	1.000	0.998	1.000	1.000	1.000	1.000
Grid: 100 × 100	1.000	1.000	1.000	1.000	1.000	1.000	1.000	0.998	1.000	1.000	1.000	1.000
Bounds: [0.10, 0.90]	1.000	1.000	1.000	1.000	1.000	1.000	1.000	0.998	1.000	1.000	0.991	0.964
Bounds: [0.02, 0.98]	1.000	1.000	1.000	1.000	1.000	1.000	1.000	0.998	1.000	1.000	1.000	1.000
Seed: 1	1.000	1.000	0.991	0.964	1.000	1.000	0.998	0.982	1.000	1.000	1.000	1.000
Seed: 2	1.000	1.000	1.000	1.000	1.000	1.000	0.998	0.980	1.000	1.000	1.000	1.000
Seed: 3	1.000	1.000	0.991	0.964	1.000	1.000	0.999	0.990	1.000	1.000	1.000	1.000
Seed: 4	1.000	1.000	1.000	1.000	1.000	1.000	0.999	0.988	1.000	1.000	1.000	1.000
Seed: 5	1.000	1.000	1.000	1.000	1.000	1.000	0.998	0.978	1.000	1.000	1.000	1.000
Minimum	1.000	1.000	0.991	0.964	0.995	0.974	0.997	0.970	0.833	0.714	0.991	0.964
Mean runtime per variation (s)	0.846		3.231		1.923		10.154		1.077		2.692	

Note: Each variation modifies a single design choice of the reference configuration; *ρ* is the Spearman and *τ* the Kendall rank correlation between the resulting ranking and the reference ranking. The penultimate row reports the minimum *ρ* and *τ* across the thirteen variations; the final row reports the mean runtime per variation (in seconds) for each dataset. The top 3 variable membership is preserved in every dataset and under every variation (3/3). The subsampling seed variations affect only the datasets in which deterministic subsampling is applied (AirQualityUCI and Meteorology); for the remaining datasets, the full sample is used and the rankings are therefore identical by construction.
